# Transgenerational epigenetic heritability for growth, body composition, and reproductive traits in Landrace pigs

**DOI:** 10.3389/fgene.2024.1526473

**Published:** 2025-01-23

**Authors:** Andre C. Araujo, Jay S. Johnson, Jason R. Graham, Jeremy Howard, Yijian Huang, Hinayah R. Oliveira, Luiz F. Brito

**Affiliations:** ^1^ Department of Animal Sciences, Purdue University, West Lafayette, IN, United States; ^2^ Livestock Behavior Research Unity, USDA-ARS, West Lafayette, IN, United States; ^3^ Smithfield Premium Genetics, Rose Hill, NC, United States

**Keywords:** ebv, epigenetics, genetic parameters, genomics, swine, variance components

## Abstract

Epigenetics is an important source of variation in complex traits that is not due to changes in DNA sequences, and is dependent on the environment the individuals are exposed to. Therefore, we aimed to estimate transgenerational epigenetic heritability, percentage of resetting epigenetic marks, genetic parameters, and predicting breeding values using genetic and epigenetic models for growth, body composition, and reproductive traits in Landrace pigs using routinely recorded datasets. Birth and weaning weight, backfat thickness, total number of piglets born, and number of piglets born alive (BW, WW, BF, TNB, and NBA, respectively) were investigated. Models including epigenetic effects had a similar or better fit than solely genetic models. Including genomic information in epigenetic models resulted in large changes in the variance component estimates. Transgenerational epigenetic heritability estimates ranged between 0.042 (NBA) to 0.336 (BF). The reset coefficient estimates for epigenetic marks were between 80% and 90%. Heritability estimates for the direct additive and maternal genetic effects ranged between 0.040 (BW) to 0.502 (BF) and 0.034 (BF) to 0.134 (BW), respectively. Repeatability of the reproductive traits ranged between 0.098 (NBA) to 0.148 (TNB). Prediction accuracies, bias, and dispersion of breeding values ranged between 0.199 (BW) to 0.443 (BF), −0.080 (WW) to 0.034 (NBA), and −0.134 (WW) to 0.131 (TNB), respectively, with no substantial differences between genetic and epigenetic models. Transgenerational epigenetic heritability estimates are moderate for growth and body composition and low for reproductive traits in North American Landrace pigs. Fitting epigenetic effects in genetic models did not impact the prediction of breeding values.

## 1 Introduction

Environmental perturbations, such as thermal stress and disease challenges, are major threats to livestock production (Lacetera, 2019). In this context, breeding more resilient animals, i.e., animals that can better cope with changing environments, has been indicated as an alternative to improve animal welfare and productivity in challenging environments (Brito et al., 2020). Genetic selection for more resilient animals using linear mixed model equations considering additive genetic effects has been in development (Tiezzi et al., 2020; Freitas et al., 2023). However, there are other non-genetic effects that may play a role in heritable variation, including microbial, cultural, and epigenetic effects (David and Ricard, 2019).

The field of epigenetics has received more attention during the past decade due to its potential to help uncover the phenotypic variation and inheritance of complex traits. However, the epigenetic term is not novel, and it was first defined in 1940s by Conrad Waddington (Waddington, 1942a; Waddington, 1942b), even before the DNA structure was known. Waddington’s first definition of epigenetics was associated with “phenotypic changes without changes in genotypes” (Waddington, 1942a; Waddington, 1942b). Nowadays, epigenetics can be defined as “the inheritance of gene expression patterns without altering the underlying DNA sequence” (Allis and Jenuwein, 2016). The epigenome is mainly composed by DNA methylations, histone modifications, chromatin remodeling, and non-coding RNA (Bird, 2002; Morris and Mattick, 2014), and can dynamically change due to a variety of external factors (exome), such as nutrition (pre- and post-natal), stress (e.g., heat, behavioral, disease), and exposure to chemicals (e.g., therapeutical drugs and toxic pollutants) or pathogens (Ibeagha-Awemu and Yu, 2021). As reviewed by Wang and Ibeagha-Awemu et al. (2021) and Ibeagha-Awemu and Yu (2021), epigenetic markers can affect various economically important traits in livestock. Therefore, it is important to investigate the amount of phenotypic variation of complex traits that can be explained by epigenetics and how this information could be used for management and breeding purposes.

Epigenetic marks are created during environmental stress and can be passed to the offspring by the germ cells and can, consequently, affect phenotypic variation in subsequent generations (Tal et al., 2010; Heard and Martienssen, 2014). Usually, epigenetic markers acquired during life by an individual would be removed during meiosis and germline reprogramming so that the embryos in the next-generation would develop based on genetic information without influence of past environment (Ibeagha-Awemu and Yu, 2021). Beyond environmental factors, epigenetic markers can be affected by intrinsic factors, such as sex and age (Wang and Ibeagha-Awemu, 2021). When the epigenic mark reset fails, some epigenetic marks are inherited and can affect the next generations (Heard and Martienssen, 2014). Intergenerational epigenetic effects can impact phenotypic variation up to two generations on the dam side or one generation on the sire side after exposure to the stressors, while transgenerational epigenetic effects influence phenotypic variability in further generations even without additional environmental stressors, when they can remain or be lost (Heard and Martienssen, 2014).

Recent studies have aimed to model the effect of stressors (e.g., heat stress) on future performance and next generations in genetic evaluations to capture epigenetic effects (Kipp et al., 2021; Weller et al., 2021). For instance, Kipp et al. (2021) used random regression models with Legendre orthogonal polynomials to model time-lagged temperature humidity index (THI) experienced by dairy cattle animals during the last week before birth (pre-natal or in uterus heat stress) in a genetic model. Weller et al. (2021) tested the hypothesis of transmission of transgenerational heat stress effects by evaluating the effect of the exposure of F0 (great grandmother) dairy cows during pregnancy over a F3 (great-granddaughter) generation of cows. These studies used indirect approaches to determine the effect of stressors on the phenotypic variation of animals in an intergenerational (Kipp et al., 2021) and trans-generational way (Weller et al., 2021), i.e., they did not formally include an epigenetic effect in the statistical genetic models.

Previously, Varona et al. (2015) proposed an approach for directly accounting for the transgenerational epigenetic effects in the animal models commonly used for livestock genetic evaluations. The authors proposed to include the transgenerational epigenetic effect as an additional random effect in the animal models, with the inverse of the epigenetic relationship matrix (**Λ** ^**−1**^) used as the covariance structure for the epigenetic effect at the individual level. The epigenetic relationship matrix (**Λ**) and its inverse were derived by Varona et al. (2015) using the theory developed by Tal et al. (2010). The advantages of using the Varona’s et al. (2015) method are: 1) it allows the estimation of the proportion of phenotypic variance explained by the transgenerational epigenetic effects, termed as transgenerational epigenetic heritability; 2) it is an epigenetic effect associated with an overall response to environment/stress instead of only accounting for specific stressors (e.g., heat stress); 3) it provides epigenetic solutions for all animals included in the pedigree file; and, 4) it is based on routinely-recorded datasets as opposed to generating additional datasets such as whole-genome bisulfite sequencing for assessing DNA methylation patterns. However, the method is based on strong assumptions, such as the independence of epigenetic, genetic, and residual effects. Few studies have applied the Varona’s et al. (2015) method in the literature (Varona et al., 2015; Paiva et al., 2018a; Paiva et al., 2018b), and none to our knowledge have used pig datasets. There are well-established reports of lifelong negative postnatal effects of prenatal (*in utero*) heat stress for production and reproduction traits in pigs (Johnson and Baumgard, 2019), but the effect of epigenetics on phenotypic variation in complex traits in pigs is still unknown. Therefore, the primary study objectives were to estimate transgenerational epigenetic heritability, determine the percentage of the reset and transmissibility rate of epigenetic marks, as well as to estimate genetic parameters and predict breeding values based on statistical models fitting exclusively genetic or genetic and epigenetic effects for growth, body composition, and reproductive traits in North American Landrace pigs.

## 2 Materials and methods

No ethical and animal care approval was needed for this study because all the data used was previously collected and provided by commercial breeding operations.

### 2.1 Traits and phenotypic quality control

Datasets from five nucleus herds located in North Carolina and Texas (USA) were provided by Smithfield Premium Genetics (SPG; Rose Hill, NC, United States) and consisted of records from purebred Landrace pigs. Measurements were collected between the years 2014–2019. The five traits included in this study were categorized as: 1) growth: birth weight (BW) and weaning weight (WW); 2) body composition: off-test backfat (BF); and 3) reproduction traits: total number of piglets born (TNB) and number of piglets born alive (NBA).

Records outside of an interval of 3.5 standard deviations (SD) from the mean were removed from further analyses. Observations from animals above the sixth parity or sows with less than 3 or more than 20 piglets per litter were also removed due to lower incidence. Animals with phenotypic observations but not present in the pedigree file and animals whose pedigrees were not complete for at least four generations were also removed from the analyses. This quality control was done to keep only relevant information for the variance component estimation analyses (Nilforooshan and Saavedra-Jiménez, 2020), especially for estimating epigenetic effects that require deeper knowledge of the genealogy and more complex partition of the phenotypic variance (Tal et al., 2010). The “optiSel” package in R (Wellmann, 2019) was used to check for erroneous pedigree entries and pedigree completeness. After the phenotypic data editing, the contemporary groups (CG) were defined by the concatenation of birth year, month, and farm for the growth and body composition traits and farrow year, month, and farm for the reproductive traits. The CG were defined based on the trait groups to better capture animals that would move together and share similar management practices (i.e., contemporaries). The number of animals and the descriptive statistics for all traits, after quality control, as well as the number of CG are included in Table 1. A minimum number of five animals was set for each level of the CG effect as well as for all other fixed effects included in the models (described later).

**TABLE 1:** Descriptive statistics for birth weight (BW), weaning weight (WW), backfat (BF), total number of piglets born (TNB), and number of piglets born alive (NBA) in North American Landrace pigs.

Variable^a^	Trait				
	BW (kg)	WW (kg)	BF (inch)	TNB (n)	NBA (n)
Individuals	10,862	10,862	10,862	5,210	5,210
Records	10,862	10,862	10,862	10,051	10,051
SD	0.34	1.73	0.14	3.45	3.26
CG	138	138	138	45	45

^a^ Number of phenotyped individuals (Individuals), records (Records), standard deviation (SD), and contemporary groups (CG) after the quality control.

### 2.2 Pedigree and genotypes

After pedigree quality control, the pedigree consisted of 17,794 animals, spanning a maximum of 12 generations, with a mean (SD) pedigree completeness index of 0.84 (0.33) for growth and body composition traits, while for reproductive traits, the pedigree comprised 8,390 animals, spanning 11 generations, with a pedigree completeness index of 0.83 (0.29). The “optiSel” package in R (Wellmann, 2019) was also used to calculate the pedigree statistics shown above. The RENUMF90 package from the BLUPF90+ family of programs (Misztal et al., 2018) was used to renumber the pedigree and keep up to four generations back from the animals with phenotypic and/or genomic information to reduce the pedigree and, consequently, time to reach convergence and memory usage during the analyses (Nilforooshan and Saavedra-Jiménez, 2020). Previous analyses tracing up to 20 generations back in the pedigree using RENUMF90 (Misztal et al., 2018) did not affect the estimates (results not shown).

Genotypes were available for 12,759 individuals genotyped with a Porcine SNP50K Bead Chip (Illumina, San Diego, CA, United States). The quality control (QC) was performed using the PREGSF90 software (Misztal et al., 2018) to remove samples and SNPs with call rate lower than 0.90 and SNPs with minor allele frequency lower than 0.05, SNPs with a difference between expected and observed heterozygous greater than 0.15, and SNPs located in non-autosomal chromosomes or with unknown genomic positions. After QC, 12,759 individuals and 34,524 SNPs remained for further analyses.

### 2.3 Transgenerational epigenetic variance and heritability estimation

The transgenerational epigenetic component of covariance between relatives was derived based on the method proposed by Tal et al. (2010). The main assumption made by the authors was that the covariances between close relatives change when epigenetic marks are being transmitted across generations. Later, Varona et al. (2015) used this theory to derive the **Λ** and **Λ** ^**−1**^. The **Λ** matrix is calculated using the auto-recursive parameter λ , in which $\lambda =0.5\left(1-\nu \right)$ , where ν is the reset coefficient, defined as the probability that the epigenetic state of the genome changes from one generation to the next. In brief, Tal et al. (2010) proposed a reset coefficient ( ν ) of the epigenetic marks and its complement ( 1−λ ) as the transmissibility of the epigenetic marks, which weights the covariance between relatives in the presence of factors that can cause epigenetic changes. A Python function (see Additional file 1) was implemented to calculate the lower triangular and diagonal portion of **Λ** ^**−1**^ directly in the long format to estimate the transgenerational epigenetic component of variance ( $\sigma_{\xi }^{2}$ ) and heritability ( $h_{\xi }^{2}$ ) using the BLUPF90 family programs (Misztal et al., 2018). The Phyton function developed in this study was based on the algorithm proposed by Varona et al. (2015), which assumed the same epigenetic variance across generations and between sexes. These assumptions allow the calculation of the epigenetic heritability (or epigenetic ratio) as $h_{\xi }^{2}=\frac{\hat{\sigma_{\xi }^{2}}}{\hat{\sigma_{y}^{2}}}$ , where $\hat{\sigma_{y}^{2}}$ is the phenotypic variance, i.e., is the sum of all variance components from the model used to evaluate each trait.

### 2.4 Statistical methods

Model building was conducted before fitting the epigenetic models. The systematic effects (presented later) were variables statistically significant at 5% probability in a linear model from a set of environmental (non-genetic) variables. Once the systematic effects were defined for each trait, the set of random effects to be used in the genetic models were chosen under the Restricted Maximum Likelihood (REML) (Patterson and Thompson, 1971) approach using the Average Information algorithm (AI-REML) (Gilmour et al., 1995) and the Akaike Information Criterion (AIC) (Akaike, 1974). The rank for the genetic models using different sets of random effects is presented in Supplementary Material 2: Supplementary Table S1, and the best genetic model (lowest AIC with meaningful estimates of the genetic parameters, i.e., within or close to previous estimates for the parameter and trait) for the evaluated traits were:

y=Xb+Zu+Zm+Sq+e (1)

y=Xb+Zu+Zm+e (2)

y=Xb+Zu+Wpe+e (3)

where Equations 1–3 are the genetic models for the growth (BW and WW), back fat thickness (BF), and reproductive (TNB and NBA) traits, respectively; y is the vector of the phenotypic records for each trait; b is the vector of systematic effects (for BW: gender, birth parity, and CG; WW: gender, birth parity, CG, and weaning age and birth weight as covariates; BF: gender, birth parity, CG, and off-test age as covariate; TNB and NBA: farrowing age as linear and quadratic covariates, CG, and parity); u is the vector of random direct additive genetic effects; m is the vector of random maternal additive genetic effects; q is the vector of random common litter environment effects; pe is the vector of random permanent environmental effects; and **e** is the vector of random residuals. X , Z , S , and W are the incidence matrices for b , u , m , q , and pe , respectively. BW was used as a covariate in the WW to account for the starting point of the growth trajectory in this trait. The AIREMLF90 software (Misztal et al., 2018) was used to estimate variance components under the following assumptions for each genetic model:

$$\left[\begin{array}{c} \begin{array}{c} u\\ m\\ q \end{array}\\ e \end{array}\right]∼N\left(\left[\begin{array}{c} 0\\ 0\\ \begin{array}{c} 0\\ 0 \end{array} \end{array}\right],\left[\begin{array}{cccc} A\sigma_{u}^{2} & A\sigma_{u,m} & 0 & 0\\ A\sigma_{u,m} & A\sigma_{m}^{2} & 0 & 0\\ 0 & 0 & I\sigma_{q}^{2} & 0\\ 0 & 0 & 0 & I\sigma_{e}^{2} \end{array}\right]\right)$$ (4)

$$\left[\begin{array}{c} \begin{array}{c} u\\ m\\ q \end{array}\\ e \end{array}\right]∼N\left(\left[\begin{array}{c} 0\\ 0\\ \begin{array}{c} 0\\ 0 \end{array} \end{array}\right],\left[\begin{array}{cccc} A\sigma_{u}^{2} & 0 & 0 & 0\\ 0 & A\sigma_{m}^{2} & 0 & 0\\ 0 & 0 & I\sigma_{q}^{2} & 0\\ 0 & 0 & 0 & I\sigma_{e}^{2} \end{array}\right]\right)$$ (5)

$$\left[\begin{array}{c} u\\ m\\ e \end{array}\right]∼N\left(\left[\begin{array}{c} 0\\ 0\\ 0 \end{array}\right],\left[\begin{array}{ccc} A\sigma_{u}^{2} & A\sigma_{u,m} & 0\\ A\sigma_{u,m} & I\sigma_{m}^{2} & 0\\ 0 & 0 & I\sigma_{e}^{2} \end{array}\right]\right)$$ (6)

$$\left[\begin{array}{c} u\\ pe\\ e \end{array}\right]∼N\left(\left[\begin{array}{c} 0\\ 0\\ 0 \end{array}\right],\left[\begin{array}{ccc} A\sigma_{u}^{2} & 0 & 0\\ 0 & I\sigma_{\text{pe}}^{2} & 0\\ 0 & 0 & I\sigma_{e}^{2} \end{array}\right]\right)$$ (7)

where the assumption (Equation 4) was used for BW, Equation 5 for WW, Equation 6 for BF, and Equation 7 for TNB and NBA. The pedigree-based relationship matrix ( A ) was used to model the covariances in u and m in the genetic analysis, and the identity ( I ) matrix was used for the q , pe , and e . The $\sigma_{u}^{2}$ , $\sigma_{m}^{2}$ , $\sigma_{q}^{2}$ , $\sigma_{\text{pe}}^{2}$ , and $\sigma_{e}^{2}$ are the variance components for u , m , q , pe , and e , respectively, and $\sigma_{u,m}$ is the covariance between **u** and **m**. The genetic models, i.e., models using the A as covariance structure for the additive genetic effects, will be called BLUP models from now on.

The A matrix was replaced by the H matrix in the BLUP models when the genomic information was included in the estimation of genomic-based variance components, i.e., single-step genomic BLUP models (ssGBLUP) (Legarra et al., 2009; Christensen and Lund, 2010). This H is the matrix that combines the pedigree and genomic information, and its inverse ( $H^{-1}$ ) was computed directly as (Aguilar et al., 2010):

$$H^{-1}=A^{-1}+\left[\begin{array}{cc} 0 & 0\\ 0 & \tau {\left(\alpha G+\beta A_{22}\right)}^{-1}-\omega A_{22}^{-1} \end{array}\right]$$ (8)

where $A^{-1}$ , $A_{22}$ , $A_{22}^{-1}$ , and G are the inverse of the pedigree relationship matrix, part of the A related to the genotyped animals, its inverse, and the genomic relationship matrix, respectively (Equation 8). The G was computed as in method 1 proposed by VanRaden (2008). The scaling parameters ( τ and ω ) were equal to 1.00, and the blending parameters α and β were equal to 0.95 and 0.05, respectively.

The epigenetic model for each trait was obtained by expanding the BLUP models for each trait including the transgenerational epigenetic effect, which will be called Epi-BLUP. In summary, the term Zξ was included in the models (Equations 1–3), which became:

y=Xb+Zu+Zm+Sq+Zξ+e (9)

y=Xb+Zu+Zm+Zξ+e (10)

y=Xb+Zu+Wpe+Zξ+e (11)

where Equations 9–11 are the epigenetic models for growth (BW and WW), backfat thickness (BF), and reproductive (TNB and NBA) traits, respectively; ξ is the vector of epigenetic effects, with $\xi \;∼N\left(0,\Lambda \sigma_{\xi }^{2}\right)$ assumed to be uncorrelated to all the other random effects, as proposed by Tal et al. (2010) and Varona et al. (2015); all the other effects were previously described. To estimate the transgenerational epigenetic heritability, the best epigenetic model, i.e., the model that presented the best λ , was defined first because the reset and transmissibility of the epigenetic markers are population-specific parameters. In this sense, a grid search ranging from 0.05 to 0.45 by 0.05 (totaling nine epigenetic models for each trait) was evaluated for λ in the creation of **Λ** ^−1^ that was fitted directly in the epigenetic models. The H matrix (Legarra et al., 2009) was also used to model the additive genetic relationships in the epigenetic models to study the impact of genomic information in these models, i.e., epigenetic models including genomic information (Epi-ssGBLUP) and not epigenomic models (e.g., using whole-genome DNA methylation).

### 2.5 Model comparison

Even though both REML-based and Bayesian inference-based methods should converge to the same population parameters, there are differences in the statistical properties of these methods, which are important to consider, especially if the models are complex (Gianola and Fernando, 1986). In this context, we have estimated variance components based on BLUP, ssGBLUP, Epi-BLUP, and Epi-ssGBLUP models using Bayesian inference. Bayesian inference was used in this step because the epigenetic models have a complex covariance structure and this method is more robust to model complexity (Gianola and Fernando, 1986).

The best Epi-BLUP or Epi-ssGBLUP model for each trait, i.e., with the best λ , under Bayesian inference was chosen using the Deviance Information Criterion (DIC; lower values indicate better model fit) (Spiegelhalter et al., 2002). Maximum likelihood estimates from the AI-REML runs were used as starting values in the Bayesian inference to improve the sampling process (Harms and Roebroeck, 2018) as poor starting values tend to slow model convergence (Raftery and Lewis, 1992). The THRGIBBS1F90 software (Misztal et al., 2018) was used to estimate variance components considering all traits as linear traits. Initial values for size, burnin, and interval in the MCMC chain were defined as 100,000, 10,000, and 10, respectively, for all traits and models, and these values were increased if convergence was not achieved based on the author’s experience and final values are presented in the results section. Convergence was verified based on the Geweke diagnostic (Geweke, 1992) with 5% probability, a stationarity test (Heidelberger and Welch, 1983) criterion, and visual inspection. The convergence tests and visual inspection of the chains were done using the R software (R Language, 2012) with the “boa” package (Smith, 2007).

### 2.6 Evaluating prediction results

After estimating the variance components, the solutions of the mixed model equations for the best fit models were evaluated. The purposes of these analyses were mainly to: 1) investigate the association between the solutions of the additive, maternal, and permanent environment effects in a genetic or genomic model including the transgenerational epigenetic effects; 2) assess the changes in the solutions by including the transgenerational epigenetic effects; and 3) evaluate the prediction accuracy, bias, and dispersion of the breeding values in young non-phenotyped individuals when including the transgenerational epigenetic effects in the models.

The association between the additive, maternal, and permanent environment effects when including the transgenerational epigenetic effects in the models was evaluated using a Pearson correlation between the solutions considering all animals used for variance component estimation (please see the sections *Traits and dataset edits* and *Pedigree and Genotypes*). Changes in the solutions when including the transgenerational epigenetic effects were evaluated using a paired *t*-test with a significance level of 0.05% for the difference between the solutions, also considering all animals used for variance component estimation.

The prediction accuracy, bias, and dispersion were investigated using the Linear Regression method (LR) (Legarra and Reverter, 2018). In brief, a set of animals born in 2019 (N = 571) chosen among the ones used for the estimation of variance components had their phenotypic records masked for BW, WW, and BF, and were considered as the focal animals (young non-phenotyped selection candidates). In the case of TNB and NBA, the focal animals for the LR method were born in 2017 (N = 933), because this was the last year with phenotyped animals for the reproductive traits. After defining the focal animals, the whole and partial datasets for applying the LR method were created. The whole dataset included all animals and phenotypes used for variance component estimation in the prediction, while in the partial dataset the phenotypes for the focal animals were removed, so that their estimated breeding values (EBV) were obtained based on the relationships with the remaining animals with phenotypic records. In the end, the prediction accuracy, bias, and dispersion of the EBV for the focal animals were calculated as:

$$\text{Accuracy}=\sqrt{\frac{\text{cov}\left(\text{EB}V_{W},\text{EB}V_{I}\right)}{\left(1-\bar{F}\right){\hat{\sigma }}_{u}^{2}}}$$ (12)

$$\text{Bias}=\text{ave}\left(\hat{u_{\text{EB}V_{I}}}\right)-\text{ave}\left(\hat{u_{\text{EB}V_{W}}}\right)$$ (13)

$$\text{Dispersion}=\left(\frac{\text{cov}\left(\text{EB}V_{W},\text{EB}V_{I}\right)}{\text{var}\left(\text{EB}V_{I}\right)}\right)-1$$ (14)

where $\text{cov}\left(\text{EB}V_{W},\text{EB}V_{I}\right)$ is the covariance between the EBV in the whole ( $\text{EB}V_{W}$ ) and incomplete, i.e., partial, ( $\text{EB}V_{I}$ ) datasets, $\bar{F}$ is the average inbreeding (pedigree or genomic), $\text{ave}\left(\right)$ represent the arithmetic average function, $\hat{u_{\text{EB}V_{W}}}$ and $\hat{u_{\text{EB}V_{I}}}$ are the predicted EBV in the whole and incomplete datasets, respectively, and $v\text{ar}\left(\text{EB}V_{I}\right)$ is the variance of the EBV in the incomplete datasets (Equations 12–14). The other equation components were previously described.

## 3 Results

### 3.1 Model comparison

The Epi-BLUP model presented a better fit (lower DIC) compared to BLUP for BW, WW, and BF (Table 2). λ = 0.05 provided the lowest DIC for BW and WW in the Epi-BLUP models (10,387.47 and 44,350.08, respectively), and λ = 0.10 was the best for BF (−30,458.31), which were lower than the DIC of the BLUP models for these traits (21,258.82, 52,803.60, and −17,142.00, respectively). Epi-BLUP models with λ = 0.05 provided slightly lower DIC for TNB and NBA (52,651.09 and 51,609.69, respectively) compared to the BLUP model (52,651.31 and 51,610.34, respectively), suggesting that the Epi-BLUP models would be a better choice for these traits as well.

**TABLE 2:** Deviance Information Criteria (DIC) of genetic and epigenetic models including or not genomic information in the relationship matrix for birth weight (BW), weaning weight (WW), backfat thickness (BF), total number of piglets born (TNB), and number of piglets born alive (NBA) in North American Landrace pigs.

Model	BW	WW	BF	TNB	NBA
BLUP^a^	21,258.82	52,803.60	−17,142.00	52,651.31	51,610.34
Epi-BLUP^b^					
λ = 0.05	*10,387.47* ^e^	*44,350.08*	−28,902.60	*52,651.09*	*51,609.69*
λ = 0.10	11,147.89	48,203.01	*−30,458.31*	52,651.50	51,609.86
λ = 0.15	15,545.24	50,925.51	−29,468.32	52,652.01	51,610.20
λ = 0.20	18,905.59	51,966.21	−25,555.34	52,652.34	51,610.72
λ = 0.25	20,188.07	52,362.57	−21,887.61	52,652.59	51,611.23
λ = 0.30	20,706.73	52,553.16	−19,945.25	52,652.66	51,611.52
λ = 0.35	20,963.79	52,657.89	−18,828.57	52,652.56	51,611.28
λ = 0.40	21,113.59	52,718.82	−18,185.18	52,652.18	51,610.93
λ = 0.45	21,210.20	52,757.45	−18,119.81	52,651.63	51,610.44
ssGBLUP^c^	−453,655.16	−439,644.93	−377,412.68	−11,668.76	−101,485.82
Epi-ssGBLUP^d^					
λ = 0.05	*−1,208,421.73*	*−924,295.31*	−1,833,307.53	*−112,151.59*	*−105,368.96*
λ = 0.10	−1,186,057.16	−795,626.58	−4,113,637.44	−112,217.45	−105,341.43
λ = 0.15	−907,078.74	−640,406.81	*−6,259,876.37*	−112,154.49	−105,324.45
λ = 0.20	−686,688.81	−568,217.48	−6,182,700.78	−112,088.94	−105,240.67
λ = 0.25	−586,897.04	−529,250.64	−2,836,279.30	−104,912.69	−105,098.22
λ = 0.30	−538,938.55	−507,224.16	−1,307,419.47	−112,010.57	−105,174.30
λ = 0.35	−513,806.96	−493,565.86	−796,911.59	−111,990.23	−105,060.72
λ = 0.40	−497,627.71	−484,808.98	−577,800.96	−111,986.69	−105,050.30
λ = 0.45	−487,313.12	−479,013.17	−460,657.76	−111,959.51	−105,024.00

^a^ Best Linear Unbiased Prediction using **A** (pedigree) as the relationship matrix for the additive genetic effect.

^b^ Epigenetic model obtained by including the transgenerational epigenetic effect in the BLUP model with their respective recursive parameter ( λ ) values.

^c^ Single-step genomic BLUP obtained by replacing the **A** by the **H** (combined pedigree and genomic relationship matrix) matrix in the analyses.

^d^ Epigenetic model including genomic information obtained by expanding the ssGBLUP model by including the transgenerational epigenetic effect with their respectively λ values.

^e^ Italics DIC values indicate the lowest (best) values, in which two values were defined per trait (using or not genomic information).

When including genomic information, DIC values were lower in the best Epi-ssGBLUP models for BW, WW, BF, TNB, and NBA (−1,208,421.73, −924,295.31, −6,259,876.37, −112,151.59, −105,368.96, respectively) compared to the ssGBLUP models for these traits (−453,655.16, −439,644.93, −377,412.68, −11,668.76, −101,485.82, respectively). Similar ranks were observed between the Epi-ssGBLUP and Epi-BLUP models under Bayesian inference, with the best λ value of 0.05 for BW, WW, TNB, and NBA, except for BF, with the best λ value of 0.15.

### 3.2 Variance components and genetic parameters

The results of the genetic parameter estimation for BW are presented in Table 3. Chain size was much higher in the epigenetic models to obtain stable samples in the Markov-Monte Carlo chain (MCMC). The size of the chain for the Epi-BLUP model (biggest chain) was 1,750,000 samples with a burn-in of 1,000,000 and thin of 100, while for the BLUP model (smallest chain), these parameters were 220,000, 10,000, and 10, respectively. Small to no differences were observed among the variance components and genetic parameters between BLUP, Epi-BLUP, and ssGBLUP for BW, except for $\hat{\sigma_{e}^{2}}$ , which was much smaller in the Epi-BLUP model. In this sense, $\hat{\sigma_{P}^{2}}$ was also similar across the BLUP, Epi-BLUP, and ssGBLUP models, indicating that there was a partition of the variances in the Epi-BLUP model which resulted in the $\hat{\sigma_{\xi }^{2}}$ being extracted directly from the residuals. In the case of the Epi-ssGBLUP, there was an inflation of the $\hat{\sigma_{u}^{2}}$ and $\hat{\sigma_{m}^{2}}$ compared to the other models, which were more than two and three times larger, respectively, in the Epi-ssGBLUP. Despite the reduction in the proportion of the phenotypic variation explained by the common litter effect ( $q^{2}$ ) and $\hat{\sigma_{\xi }^{2}}$ in the Epi-ssGBLUP model, the $\hat{\sigma_{P}^{2}}$ in this model was the largest compared to all other models for BW (0.766 vs. ∼0.560). The additive direct and maternal heritability estimates followed a similar pattern of the variance components across models, but the $q^{2}$ and $\sigma_{\xi }^{2}$ , and consequently $h_{\xi }^{2}$ , were higher in the Epi-BLUP model (0.296, 0.186, and 0.330, respectively) compared to the Epi-ssGBLUP model (0.204, 0.160, and 0.209, respectively). A similar pattern of the results was observed in the WW and BF (Tables 4, 5, respectively).

**TABLE 3:** Chain parameters, posterior means, and high posterior densities of the variance components and genetic parameters estimates for the birth weight of Landrace pigs using genetic and epigenetic models with pedigree- or single-step genomic BLUP under a Bayesian approach.

Parameter^a^	BLUP^b^	Epi-BLUP^c^ ( λ=0.05 )	ssGBLUP^d^	Epi-ssGBLUP^e^ ( λ=0.05 )
MCMC chain parameters and DIC				
Size	220,000	1,750,000	225,000	625,000
Burn-in	10,000	1,000,000	126,000	225,000
Thin	10	100	10	25
DIC	21,258.821	−453,655.16	10,387.47	−1,208,421.73
Posterior means and standard deviation for the variance components and ratios				
$\sigma_{u}^{2}$	0.028 (0.011)^f^	0.022 (0.011)	0.037 (0.008)	0.077 (0.023)
$\sigma_{m}^{2}$	0.075 (0.013)	0.074 (0.013)	0.076 (0.011)	0.270 (0.039)
$\sigma_{u,m}$	−0.007 (0.011)	−0.005 (0.011)	−0.009 (0.010)	−0.014 (0.030)
$\sigma_{q}^{2}$	0.167 (0.009)	0.167 (0.009)	0.166 (0.009)	0.156 (0.009)
$\sigma_{\xi }^{2}$	—	0.186 (0.075)	—	0.160 (0.084)
$\sigma_{e}^{2}$	0.300 (0.009)	0.119 (0.074)	0.296 (0.008)	0.117 (0.082)
$\sigma_{P}^{2}$	0.563 (0.009)	0.563 (0.009)	0.567 (0.010)	0.766 (0.026)
$h_{u}^{2}$	0.050 (0.020)	0.040 (0.020)	0.065 (0.014)	0.101 (0.030)
$h_{m}^{2}$	0.132 (0.023)	0.132 (0.023)	0.134 (0.019)	0.352 (0.044)
$r_{u,m}$	−0.123 (0.227)	−0.086 (0.299)	−0.155 (0.156)	−0.083 (0.210)
$q^{2}$	0.296 (0.015)	0.296 (0.015)	0.294 (0.015)	0.204 (0.015)
$h_{\xi }^{2}$	—	0.330 (0.134)	—	0.209 (0.110)
High posterior densities for the variance components and ratios				
$\sigma_{u}^{2}$	[0.008, 0.050]	[0.003, 0.044]	[0.022, 0.053]	[0.034, 0.120]
$\sigma_{m}^{2}$	[0.049, 0.100]	[0.049, 0.100]	[0.053, 0.097]	[0.196, 0.345]
$\sigma_{u,m}$	[−0.029, 0.013]	[−0.028, 0.016]	[−0.032, 0.006]	[−0.077, 0.041]
$\sigma_{q}^{2}$	[0.149, 0.185]	[0.148, 0.185]	[0.148, 0.182]	[0.139, 0.174]
$\sigma_{\xi }^{2}$	—	[0.051, 0.309]	—	[0.014, 0.280]
$\sigma_{e}^{2}$	[0.283, 0.317]	[0.001, 0.246]	[0.281, 0.311]	[0.000, 0.248]
$\sigma_{P}^{2}$	[0.545, 0.581]	[0.544, 0.581]	[0.547, 0.586]	[0.717, 0.818]
$h_{u}^{2}$	[0.013, 0.088]	[0.005, 0.079]	[0.039, 0.094]	[0.046, 0.156]
$h_{m}^{2}$	[0.087, 0.175]	[0.086, 0.175]	[0.095, 0.168]	[0.266, 0.437]
$r_{u,m}$	[−0.550, 0.312]	[−0.629, 0.535]	[−0.514, 0.102]	[−0.491, 0.322]
$q^{2}$	[0.267, 0.327]	[0.266, 0.327]	[0.264, 0.322]	[0.174, 0.232]
$h_{\xi }^{2}$	—	[0.091, 0.552]	—	[0.017, 0.369]

^a^ Size, Burn-in, and Thin are the parameters of the Markov-Monte Carlo (MCMC) chain used to derive the posterior distribution of the parameters; DIC, deviance information criteria; $\sigma_{u}^{2}$ , $\sigma_{m}^{2}$ , $\sigma_{u,m}$ , $\sigma_{q}^{2}$ , $\sigma_{\xi }^{2}$ , $\sigma_{e}^{2}$ , $\sigma_{P}^{2}$ , $h_{u}^{2}$ , $h_{m}^{2}$ , $r_{u,m}$ , $q^{2}$ , and $h_{\xi }^{2}$ are the additive genetic, maternal genetic, covariance between additive and maternal genetic, common environment, transgenerational epigenetic, residual, and phenotypic variances, and narrow-sense (additive genetic) heritability, maternal genetic heritability, correlation between additive and maternal genetic effects, fraction of the phenotypic variance explained by the common environment effect, and transgenerational epigenetic heritability, respectively.

^b^ Best Linear Unbiased Prediction using **A** as the relationship matrix for the additive genetic effect.

^c^ Epigenetic model obtained when including the transgenerational epigenetic effect in the BLUP model with their respective recursive parameter ( λ ) values.

^d^ Single-step genomic BLUP obtained by replacing **A** for the **H** (combined pedigree and genomic relationship matrix) matrix.

^e^ Epigenetic model including genomic information obtained by expanding the ssGBLUP model by including the transgenerational epigenetic effect with their respectively λ values.

^f^ Posterior standard deviations are within parentheses.

**TABLE 4:** Chain parameters, posterior means, and high posterior densities of the variance components and genetic parameters estimates for the weaning weight of Landrace pigs using genetic and epigenetic models with pedigree- or single-step genomic BLUP under a Bayesian approach.

Parameter^a^	BLUP^b^	Epi-BLUP^c^ ( λ=0.05 )	ssGBLUP^d^	Epi-ssGBLUP^e^ ( λ=0.05 )
MCMC chain parameters and DIC				
Size	220,000	1,750,000	776,795	1,126,100
Burnin	10,000	1,002,500	250,000	550,000
Thin	10	100	25	25
DIC	52,803.60	44,350.08	−439,644.93	−924,295.31
Posterior means and standard deviation for the variance components and ratios				
$\sigma_{u}^{2}$	0.411 (0.137)^f^	0.383 (0.169)	0.399 (0.124)	1.206 (0.366)
$\sigma_{m}^{2}$	0.381 (0.093)	0.389 (0.100)	0.308 (0.081)	1.350 (0.303)
$\sigma_{q}^{2}$	2.571 (0.146)	2.562 (0.146)	2.630 (0.142)	2.540 (0.145)
$\sigma_{\xi }^{2}$	—	2.879 (1.718)	—	2.391 (1.495)
$\sigma_{e}^{2}$	5.736 (0.138)	2.885 (1.700)	5.745 (0.135)	2.956 (1.490)
$\sigma_{P}^{2}$	9.098 (0.138)	9.098 (0.138)	9.082 (0.139)	10.442 (0.293)
$h_{u}^{2}$	0.045 (0.015)	0.042 (0.018)	0.044 (0.014)	0.115 (0.034)
$h_{m}^{2}$	0.042 (0.010)	0.043 (0.011)	0.034 (0.009)	0.129 (0.026)
$q^{2}$	0.283 (0.015)	0.282 (0.015)	0.290 (0.014)	0.243 (0.016)
$h_{\xi }^{2}$	—	0.317 (0.189)	—	0.229 (0.143)
High posterior densities for the variance components and ratios				
$\sigma_{u}^{2}$	[0.172, 0.696]	[0.040, 0.677]	[0.171, 0.652]	[0.530, 1.935]
$\sigma_{m}^{2}$	[0.209, 0.556]	[0.203, 0.600]	[0.155, 0.465]	[0.740, 1.921]
$\sigma_{q}^{2}$	[2.285, 2.854]	[2.266, 2.843]	[2.359, 2.912]	[2.264, 2.832]
$\sigma_{\xi }^{2}$	—	[0.369, 5.787]	—	[0.083, 5.056]
$\sigma_{e}^{2}$	[5.459, 5.999]	[0.001, 5.325]	[5.477, 6.007]	[0.198, 5.165]
$\sigma_{P}^{2}$	[8.834, 9.377]	[8.829, 9.366]	[8.804,9.347]	[9.880, 11.020]
$h_{u}^{2}$	[0.020, 0.077]	[0.004, 0.074]	[0.020, 0.073]	[0.052, 0.183]
$h_{m}^{2}$	[0.024, 0.062]	[0.023, 0.066]	[0.018, 0.051]	[0.076, 0.180]
$q^{2}$	[0.254, 0.312]	[0.253, 0.310]	[0.263, 0.317]	[0.211, 0.273]
$h_{\xi }^{2}$	—	[0.035, 0.631]	—	[0.008, 0.485]

^a^ Size, Burn-in, and Thin are the parameters of the Markov-Monte Carlo (MCMC) chain used to derive the posterior distribution of the parameters; DIC, deviance information criteria; $\sigma_{u}^{2}$ , $\sigma_{m}^{2}$ , $\sigma_{q}^{2}$ , $\sigma_{\xi }^{2}$ , $\sigma_{e}^{2}$ , $\sigma_{P}^{2}$ , $h_{u}^{2}$ , $h_{m}^{2}$ , $q^{2}$ , and $h_{\xi }^{2}$ are the additive genetic, maternal genetic, common environment, transgenerational epigenetic, residual, and phenotypic variances, and narrow-sense (additive genetic) heritability, maternal genetic heritability, fraction of the phenotypic variance explained by the common environment effect, and transgenerational epigenetic heritability, respectively.

^b^ Best Linear Unbiased Prediction using **A** as the relationship matrix for the additive genetic effect.

^c^ Epigenetic model obtained by including the transgenerational epigenetic effect in the BLUP model with their respective recursive parameter ( λ ) values.

^d^ Single-step genomic BLUP obtained by replacing **A** for the **H** (combined pedigree and genomic relationship matrix) matrix.

^e^ Epigenetic model including genomic information obtained by expanding the ssGBLUP model by including the transgenerational epigenetic effect with their respectively λ values.

^f^ Posterior standard deviations are within parentheses.

**TABLE 5:** Chain parameters, posterior means, and high posterior densities of the variance components and genetic parameters estimates for the backfat of Landrace pigs using genetic and epigenetic models with pedigree- or single-step genomic BLUP under a Bayesian approach.

Parameter^a^	BLUP^b^	Epi-BLUP^c^ ( λ=0.10 )	ssGBLUP^d^	Epi-ssGBLUP^e^ ( λ=0.15 )
MCMC chain parameters and DIC				
Size	220,000	4,000,000	509,760	699,485
Burnin	10,000	3,000,000	250,000	350,000
Thin	10	100	25	25
DIC	−17,141.998	−30,458.31	−377,412.6797	−6,259,876.37
Posterior means and standard deviation for the variance components and ratios				
$\sigma_{u}^{2}$	0.008 (0.001)^f^	0.007 (0.001)	0.008 (0.001)	0.008 (0.001)
$\sigma_{m}^{2}$	0.001 (0.000)	0.001 (0.000)	0.001 (0.000)	0.002 (0.001)
$\sigma_{u,m}$	−0.001 (0.000)	−0.001 (0.000)	−0.001 (0.000)	0.000 (0.001)
$\sigma_{\xi }^{2}$	—	0.005 (0.002)	—	0.008 (0.001)
$\sigma_{e}^{2}$	0.008 (0.000)	0.003 (0.002)	0.008 (0.000)	0.000 (0.001)
$\sigma_{P}^{2}$	0.015 (0.000)	0.015 (0.000)	0.015 (0.000)	0.019 (0.000)
$h_{u}^{2}$	0.497 (0.041)	0.471 (0.042)	0.502 (0.037)	0.430 (0.036)
$h_{m}^{2}$	0.057 (0.019)	0.053 (0.017)	0.043 (0.014)	0.088 (0.031)
$r_{u,m}$	−0.494 (0.086)	−0.481 (0.089)	−0.534 (0.090)	0.126 (0.186)
$h_{\xi }^{2}$	—	0.336 (0.154)	—	0.436 (0.039)
High posterior densities for the variance components and ratios				
$\sigma_{u}^{2}$	[0.006, 0.009]	[0.006, 0.009]	[0.006, 0.009]	[0.007, 0.010]
$\sigma_{m}^{2}$	[0.000, 0.001]	[0.000, 0.001]	[0.000, 0.001]	[0.001, 0.003]
$\sigma_{u,m}$	[−0.002, −0.001]	[−0.002, 0.000]	[−0.002, −0.001]	[−0.001, 0.001]
$\sigma_{\xi }^{2}$	—	[0.000, 0.008]	—	[0.007, 0.009]
$\sigma_{e}^{2}$	[0.007, 0.009]	[0.000, 0.007]	[0.007, 0.009]	[0.000, 0.001]
$\sigma_{P}^{2}$	[0.015, 0.016]	[0.015, 0.016]	[0.015, 0.016]	[0.018, 0.020]
$h_{u}^{2}$	[0.418, 0.578]	[0.390, 0.554]	[0.428, 0.574]	[0.367, 0.506]
$h_{m}^{2}$	[0.023, 0.095]	[0.022, 0.089]	[0.019, 0.070]	[0.038, 0.151]
$r_{u,m}$	[−0.653, −0.318]	[−0.650, −0.299]	[−0.698, −0.351]	[−0.203, 0.462]
$h_{\xi }^{2}$	—	[0.010, 0.555]	—	[0.359, 0.506]

^a^ Size, Burn-in, and Thin are the parameters of the Markov-Monte Carlo (MCMC) chain used to derive the posterior distribution of the parameters; DIC, deviance information criteria; $\sigma_{u}^{2}$ , $\sigma_{m}^{2}$ , $\sigma_{u,m}$ , $\sigma_{\xi }^{2}$ , $\sigma_{e}^{2}$ , $\sigma_{P}^{2}$ , $h_{u}^{2}$ , $h_{m}^{2}$ , $r_{u,m}$ , and $h_{\xi }^{2}$ are the additive genetic, maternal genetic, covariance between additive and maternal genetic, residual, and phenotypic variances, and additive genetic heritability, maternal genetic heritability, correlation between additive and maternal genetic effects, and transgenerational epigenetic heritability, respectively.

^b^ Best Linear Unbiased Prediction using the **A** as the relationship matrix for the additive genetic effect.

^c^ Epigenetic model obtained by including the transgenerational epigenetic effect in the BLUP model with their respectively recursive parameter ( λ ) values below.

^d^ Single-step genomic BLUP obtained by only replacing **A** for **H** (combined pedigree and genomic relationship matrix).

^e^ Epigenetic model including genomic information obtained by expanding the ssGBLUP model by including the transgenerational epigenetic effect with their respectively λ values.

^f^ Posterior standard deviations are within parentheses.

The results of the MCMC parameters, variance components, and genetic parameters for TNB and NBA are presented in Tables 6, 7, respectively. Similar patterns were observed for the MCMC parameters, $\hat{\sigma_{u}^{2}}$ , $\hat{\sigma_{P}^{2}}$ , $h_{u}^{2}$ , and $h_{\xi }^{2}$ in the TNB and NBA compared to the growth and body composition traits. However, the source of $\hat{\sigma_{\xi }^{2}}$ in the Epi-BLUP and Epi-ssGLUP models was not due to $\hat{\sigma_{e}^{2}}$ but due to the $\hat{\sigma_{\text{pe}}^{2}}$ in the reproductive traits. There were no substantial differences in the $\hat{\sigma_{e}^{2}}$ across all models for TNB and NBA. On the other hand, $\hat{\sigma_{\text{pe}}^{2}}$ reduced in both Epi-BLUP and Epi-ssGLUP models by the same amount of variance captured by $\hat{\sigma_{\xi }^{2}}$ in TNB and NBA. Also, similar to what was observed for growth and body composition traits, the $\hat{\sigma_{P}^{2}}$ was similar to the BLUP, Epi-BLUP, and ssGBLUP estimates, suggesting that there was only repartitioning of variances in the Epi-BLUP compared to BLUP and ssGBLUP. The inflation in the $\hat{\sigma_{u}^{2}}$ , $\hat{\sigma_{P}^{2}}$ , and $h_{u}^{2}$ estimates from the Epi-ssGBLUP for the reproductive traits was also noticeable, with 3-fold increases for $\hat{\sigma_{u}^{2}}$ and $h_{u}^{2}$ , and 14% for $\hat{\sigma_{P}^{2}}$ in comparison to the ssGBLUP estimates. The posterior means of the $h_{\xi }^{2}$ for TNB and NBA in the Epi-BLUP (0.044 and 0.042, respectively) and Epi-ssGBLUP (0.033 and 0.032, respectively) were much lower compared to those for BW, WW, and BF (0.330, 0.317, 0.336, respectively, for the Epi-BLUP and 0.436, 0.229, and 0.209 for the Epi-ssGBLUP, respectively).

**TABLE 6:** Chain parameters, posterior means, and high posterior densities of the variance components and genetic parameters estimates for the total number born of Landrace pigs using genetic and epigenetic models with pedigree- or single-step genomic BLUP under a Bayesian approach.

Parameter^a^	BLUP^b^	Epi-BLUP^c^ ( λ=0.05 )	ssGBLUP^d^	Epi-ssGBLUP^e^ ( λ=0.05 )
MCMC chain parameters and DIC				
Size	100,000	7,000,000	100,000	799,225
Burn-in	10,000	1,000,000	10,000	300,000
Thin	10	100	10	25
DIC	52,651.306	52,651.091	−11,668.760	−112,151.590
Posterior means and standard deviation for the variance components and ratios				
$\sigma_{u}^{2}$	0.554 (0.127)^f^	0.541 (0.132)	0.74 (0.143)	2.549 (0.493)
$\sigma_{\text{pe}}^{2}$	1.072 (0.162)	0.590 (0.334)	0.965 (0.164)	0.442 (0.243)
$\sigma_{\xi }^{2}$	—	0.507 (0.337)	—	0.437 (0.258)
$\sigma_{e}^{2}$	9.808 (0.184)	9.802 (0.183)	9.778 (0.183)	9.771 (0.184)
$\sigma_{P}^{2}$	11.434 (0.167)	11.440 (0.166)	11.480 (0.174)	13.199 (0.412)
$h_{u}^{2}$	0.048 (0.011)	0.049 (0.012)	0.064 (0.012)	0.192 (0.032)
${\text{pe}}^{2}$	0.142 (0.013)	0.103 (0.029)	0.148 (0.013)	0.226 (0.034)
$h_{\xi }^{2}$	—	0.044 (0.029)	—	0.033 (0.020)
High posterior densities for the variance components and ratios				
$\sigma_{u}^{2}$	[0.302, 0.798]	[0.290, 0.804]	[0.488, 1.016]	[1.611, 3.529]
$\sigma_{\text{pe}}^{2}$	[0.753, 1.374]	[0.009, 1.134]	[0.659, 1.297]	[0.038, 0.865]
$\sigma_{\xi }^{2}$	—	[0.000, 1.095]	—	[0.022, 0.889]
$\sigma_{e}^{2}$	[9.449, 10.170]	[9.438, 10.150]	[9.414, 10.130]	[9.404, 10.120]
$\sigma_{P}^{2}$	[11.130, 11.780]	[11.130, 11.780]	[11.130, 11.810]	[12.390, 14.000]
$h_{u}^{2}$	[0.028, 0.070]	[0.028, 0.073]	[0.042, 0.087]	[0.130, 0.254]
${\text{pe}}^{2}$	[0.118, 0.167]	[0.048, 0.153]	[0.122, 0.176]	[0.159, 0.290]
$h_{\xi }^{2}$	—	[0.000, 0.096]	—	[0.002, 0.069]

^a^ Size, Burn-in, and Thin are the parameters of the Markov-Monte Carlo (MCMC) chain used to derive the posterior distribution of the parameters; DIC, deviance information criteria; $\sigma_{u}^{2}$ , $\sigma_{\text{pe}}^{2}$ , $\sigma_{\xi }^{2}$ , $\sigma_{e}^{2}$ , $\sigma_{P}^{2}$ , $h_{u}^{2}$ , ${\text{pe}}^{2}$ , and $h_{\xi }^{2}$ are the additive genetic, permanent environment, transgenerational epigenetic, residual, and phenotypic variances, and additive genetic heritability, fraction of the phenotypic variance explained by the permanent environment effect, and transgenerational epigenetic heritability, respectively.

^b^ Best Linear Unbiased Prediction using the **A** as the relationship matrix for the additive genetic effect.

^c^ Epigenetic model obtained by including the transgenerational epigenetic effect in the BLUP model with their respectively recursive parameter ( λ ) values below.

^d^ Single-step genomic BLUP, obtained by only replacing **A** for **H** (combined pedigree and genomic relationship matrix).

^e^ Epigenetic model including genomic information obtained by expanding the ssGBLUP model by including the transgenerational epigenetic effect with their respectively λ values.

^f^ Posterior standard deviations are within parentheses.

**TABLE 7:** Chain parameters, posterior means, and high posterior densities of the variance components and genetic parameters estimates for the number of piglets born alive of Landrace pigs using genetic and epigenetic models with pedigree- or single-step genomic BLUP under a Bayesian approach.

Parameter^a^	BLUP^b^	Epi-BLUP^c^ ( λ=0.05 )	ssGBLUP^d^	Epi-ssGBLUP^e^ ( λ=0.05 )
MCMC chain parameters and DIC				
Size	100,000	7,000,000	300,000	944,125
Burn-in	10,000	1,000,000	10,000	350,000
Thin	10	100	10	25
DIC	51,610.338	51,609.693	−101,485.824	−105,368.962
Posterior means and standard deviation for the variance components and ratios				
$\sigma_{u}^{2}$	0.558 (0.128)^f^	0.543 (0.131)	0.639 (0.111)	2.340 (0.455)
$\sigma_{\text{pe}}^{2}$	0.878 (0.151)	0.471 (0.279)	0.803 (0.143)	0.364 (0.205)
$\sigma_{\xi }^{2}$	—	0.435 (0.284)	—	0.380 (0.218)
$\sigma_{e}^{2}$	8.871 (0.167)	8.863 (0.166)	8.87 (0.166)	8.836 (0.166)
$\sigma_{P}^{2}$	10.307 (0.151)	10.311 (0.150)	10.311 (0.152)	11.920 (0.379)
$h_{u}^{2}$	0.054, (0.012)	0.053 (0.012)	0.062 (0.010)	0.195 (0.032)
${\text{pe}}^{2}$	0.139 (0.013)	0.098 (0.029)	0.140 (0.013)	0.226 (0.034)
$h_{\xi }^{2}$	—	0.042 (0.028)	—	0.032 (0.019)
High posterior densities for the variance components and ratios				
$\sigma_{u}^{2}$	[0.311, 0.806]	[0.296, 0.803]	[0.418, 0.864]	[1.465, 3.227]
$\sigma_{\text{pe}}^{2}$	[0.585, 1.162]	[0.002, 0.935]	[0.511, 1.073]	[0.025, 0.729]
$\sigma_{\xi }^{2}$	—	[0.000, 0.928]	—	[0.026, 0.778]
$\sigma_{e}^{2}$	[8.537, 9.195]	[8.534, 9.184]	[8.550, 9.197]	[8.512, 9.163]
$\sigma_{P}^{2}$	[10.000, 10.590]	[10.020, 10.600]	[10.024, 10.614]	[11.204, 12.673]
$h_{u}^{2}$	[0.031, 0.078]	[0.029, 0.078]	[0.041, 0.083]	[0.132, 0.258]
${\text{pe}}^{2}$	[0.115, 0.164]	[0.045, 0.148]	[0.114, 0.164]	[0.162, 0.292]
$h_{\xi }^{2}$	—	[0.000, 0.090]	—	[0.002, 0.066]

^a^ Size, Burn-in, and Thin are the parameters of the Markov-Monte Carlo (MCMC) chain used to derive the posterior distribution of the parameters; DIC, deviance information criteria; $\sigma_{u}^{2}$ , $\sigma_{\text{pe}}^{2}$ , $\sigma_{\xi }^{2}$ , $\sigma_{e}^{2}$ , $\sigma_{P}^{2}$ , $h_{u}^{2}$ , ${\text{pe}}^{2}$ , and $h_{\xi }^{2}$ are the additive genetic, permanent environment, transgenerational epigenetic, residual, and phenotypic variances, and additive genetic heritability, fraction of the phenotypic variance explained by the permanent environment effect, and transgenerational epigenetic heritability, respectively.

^b^ Best Linear Unbiased Prediction using the **A** as the relationship matrix for the additive genetic effect.

^c^ Epigenetic model obtained by including the transgenerational epigenetic effect in the BLUP model with their respectively recursive parameter ( λ ) values below.

^d^ Single-step genomic BLUP obtained by only replacing **A** for **H** (combined pedigree and genomic relationship matrix).

^e^ Epigenetic model including genomic information obtained by expanding the ssGBLUP model by including the transgenerational epigenetic effect with their respective λ values.

^f^ Posterior standard deviations are within parentheses.

### 3.3 Association between variance components and genetic parameters

**FIGURE 1:**
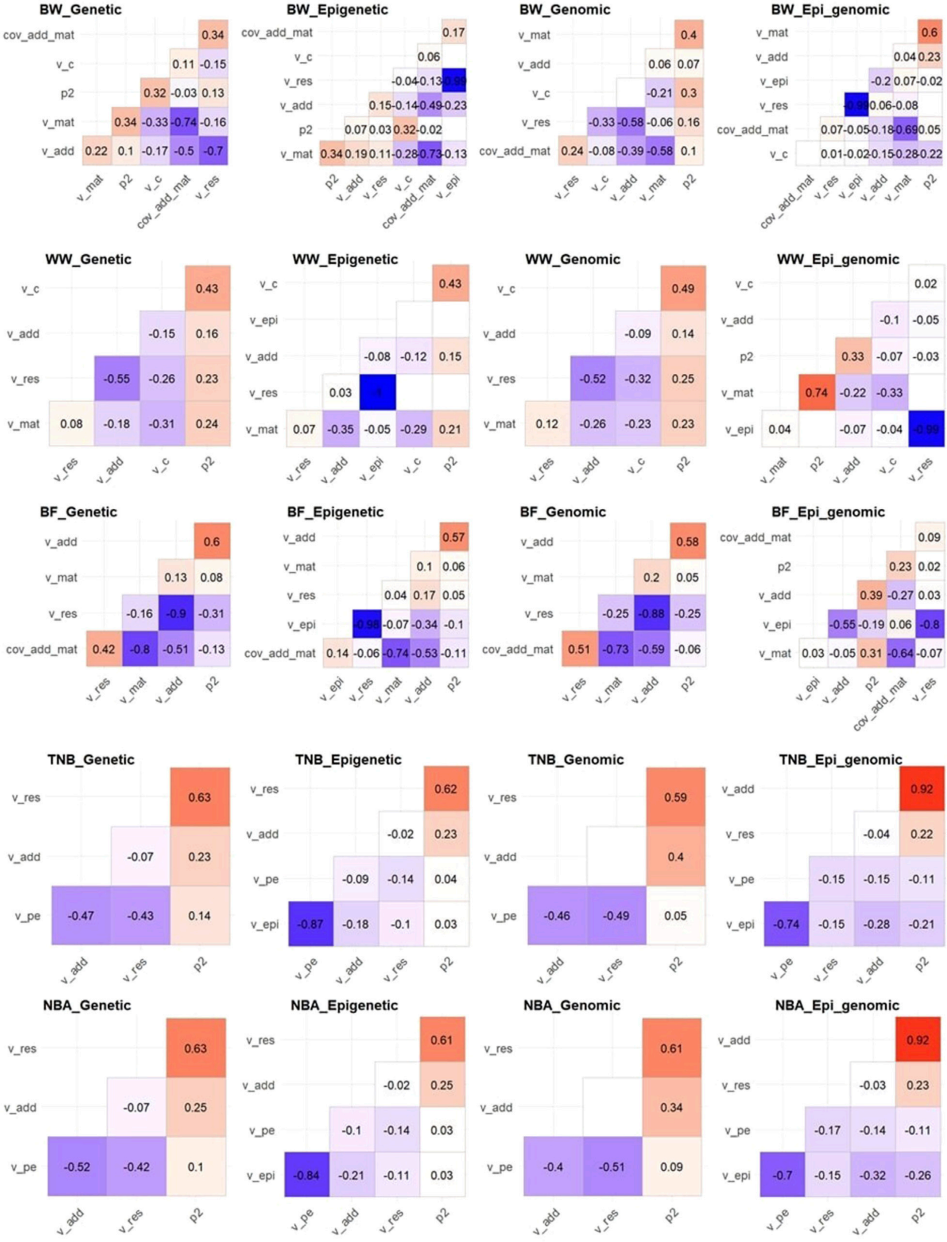
Correlation among posterior samples of the parameters used in the genetic, epigenetic, genomic, and epigenetic models including genomic information (Epi_genomic) for birth weight (BW), weaning weight (WW), backfat (BF), total number of piglets born (TNB), and number of piglets born alive (NBA) in Landrace pigs. v_add, v_mat, v_c, v_pe, v_epi, v_res, p2, cov_add_mat = additive genetic, maternal genetic, common environment, permanent environment, transgenerational epigenetic, residual, and phenotypic variances, and covariance between additive genetic and maternal genetic effects, respectively. The heritability and ratios had the same pattern of their respective variance components and were omitted for simplicity. Blank squares mean non-significant correlation coefficient at 5% of probability.

Despite the assumptions of independence among the random effects in all models, we observed that the posterior samples are correlated in most cases (Figure 1). The magnitude of the correlations among the variance components tended to be smaller in the Epi-BLUP and Epi-ssGBLUP in comparison to the estimates from the BLUP and ssGBLUP models for all traits, respectively, except for $\sigma_{\xi }^{2}$ , which was not fitted in these later models. Similar correlations were observed between the variance components in the BLUP and ssGBLUP models. The $\sigma_{\xi }^{2}$ was highly negatively correlated to $\sigma_{e}^{2}$ in the BW and WW for both Epi-BLUP and Epi-ssGBLUP models ( r ≤ −0.99), while only a low negative correlation was observed between $\sigma_{\xi }^{2}$ and $\sigma_{u}^{2}$ for these traits (−0.23 ≤ r ≤ −0.07). For BF, $\sigma_{\xi }^{2}$ was also highly negatively correlated to $\sigma_{e}^{2}$ with Epi-BLUP ( r = −0.98), while a high negative correlation was observed for Epi-ssGBLUP ( r = −0.80), and a negative moderate correlation was observed between $\sigma_{\xi }^{2}$ and $\sigma_{u}^{2}$ in the Epi-BLUP and Epi-ssGBLUP models (−0.34 and −0.55, respectively). A low negative correlation was observed between $\sigma_{\xi }^{2}$ and $\sigma_{e}^{2}$ for TNB and NBA in both Epi-BLUP and Epi-ssGBLUP models (−0.15 ≤ r ≤ −0.10), while a moderate to high negative correlation was observed between $\sigma_{\xi }^{2}$ and $\sigma_{\text{pe}}^{2}$ (−0.87 ≤ r ≤ −0.70), and a low to moderate negative correlation was observed for $\sigma_{\xi }^{2}$ and $\sigma_{u}^{2}$ (−0.32 ≤ r ≤ −0.18).

### 3.4 Prediction results

**FIGURE 2:**
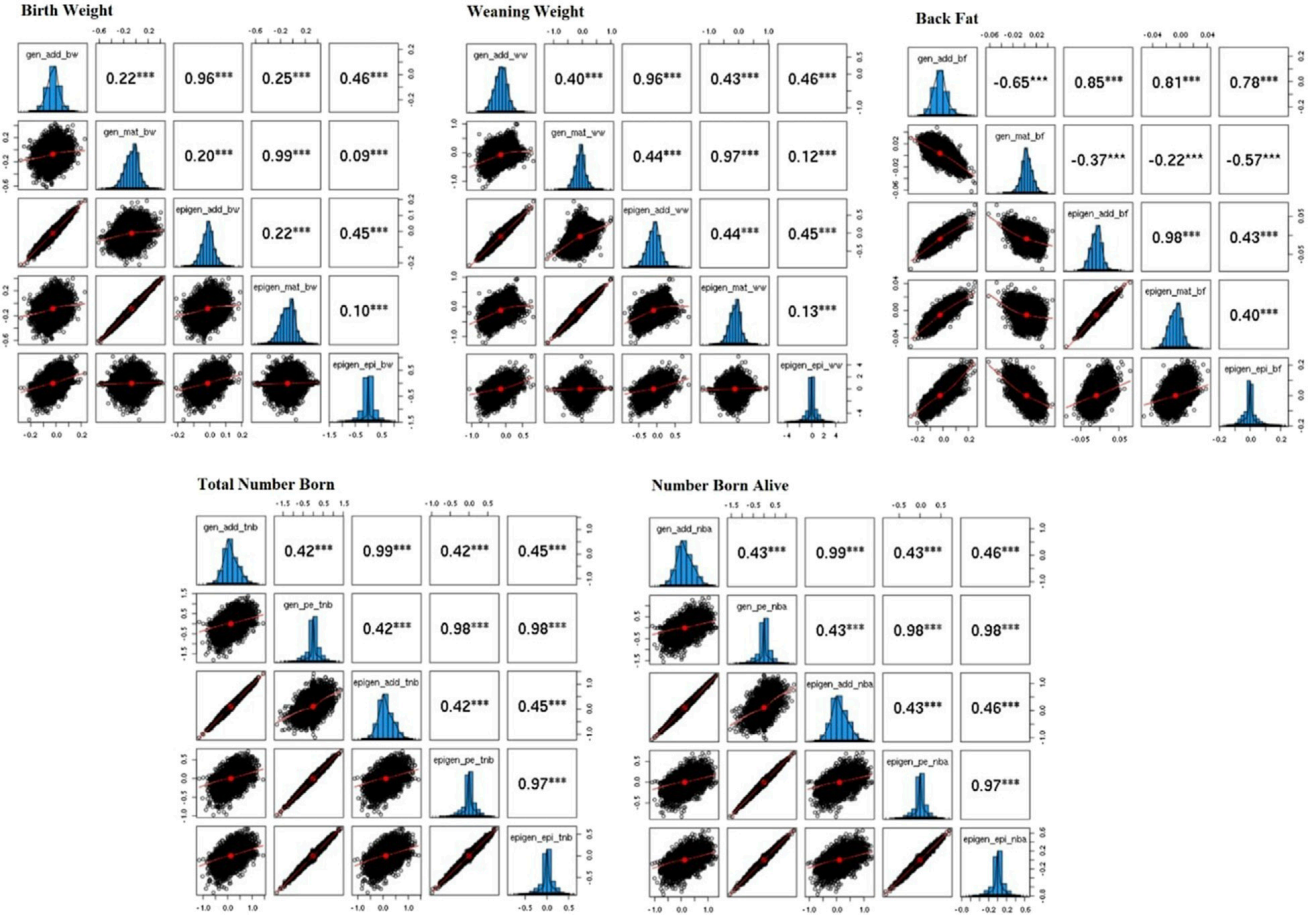
Correlation among solutions from mixed model equations using a genetic and epigenetic model for the birth weight, weaning weight, backfat, total number of piglets born, and number of piglets born alive in Landrace pigs. gen_add_bw, gen_mat_bw, epi_add_bw, epi_mat_bw, epi_epi_bw = solutions for the direct additive genetic effect from genetic model, solutions for the maternal genetic effect from genetic model, solutions for the direct additive genetic effect from epigenetic model, solutions for the maternal genetic effect from epigenetic model in the birth weight, respectively; same pattern was employed for the other traits changing the trait suffix for ww, bf, tnb, and nba, for weaning weight, back fat, total number born, and number born alive, respectively, the only exception was for total number born and number born alive, that did not have maternal genetic effects and presented permanent environment effect, called as gen_pe_tnb, epi_pe_tnb for the solutions for the permanent environment effects for genetic and epigenetic models for total number born, respectively, same pattern was applied for number born alive.

The correlations between solutions for u , m , and ξ for BW, WW, and BF, and u , pe , and ξ for TNB and NBA in the BLUP and Epi-BLUP models are presented in Figure 2. Only the correlations among solutions in the genetic and epigenetic models with the best λ , not including genomic information, are presented here. High correlations were observed between the direct additive genetic solutions in BLUP and Epi-BLUP models for all traits, in which the lowest correlation of 0.85 was observed for BF and all others were above 0.96. The correlation for the maternal genetic solutions between BLUP and Epi-BLUP models was low for BF (−0.22) and high for BW and WW (0.99 and 0.97, respectively). In the case of TNB and NBA, the permanent environment solutions were highly correlated (0.98) between BLUP and Epi-BLUP models.

**FIGURE 3:**
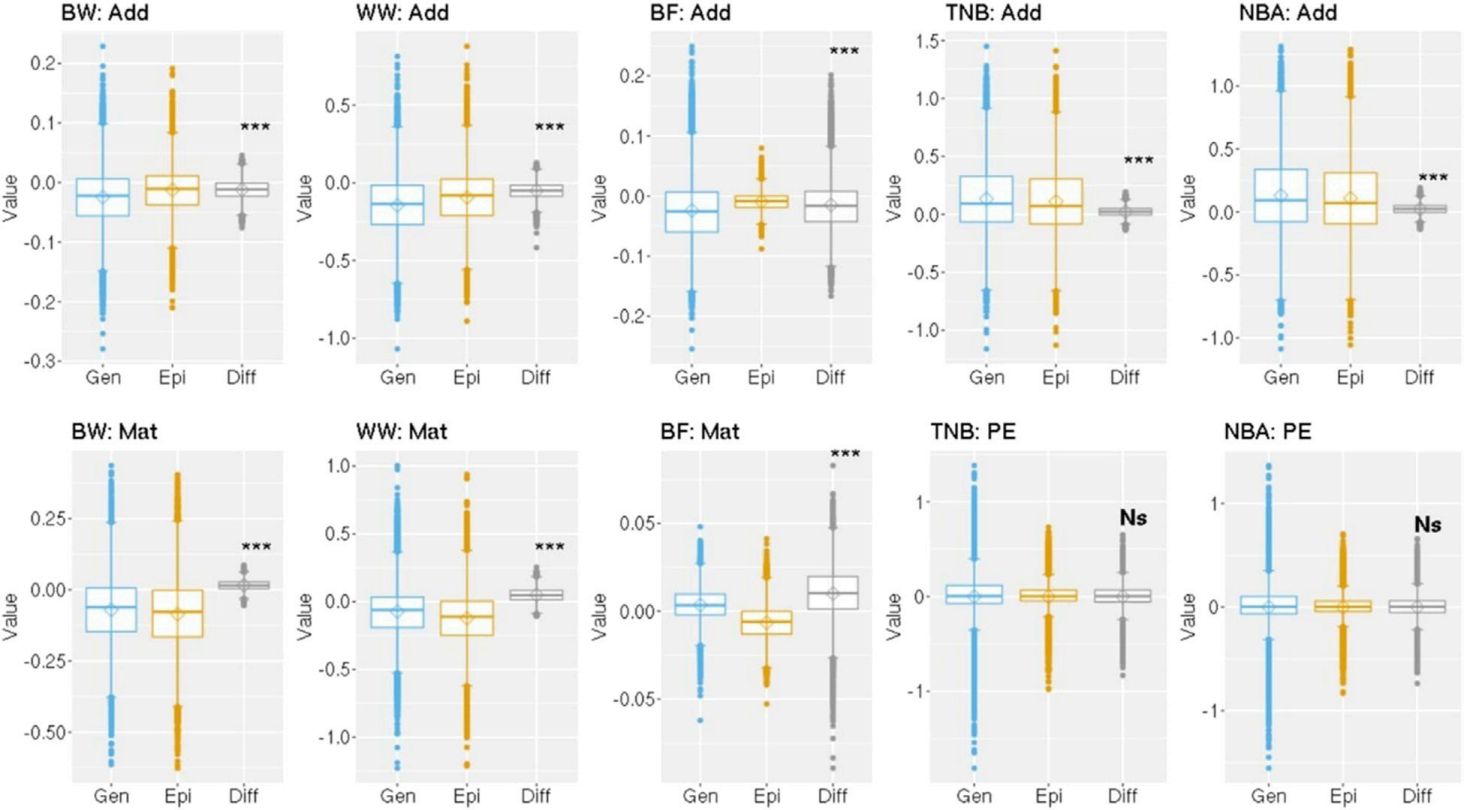
Distribution of the solutions and difference among solutions between the genetic and epigenetic models for the random effects used to analyze birth weight (BW), weaning weight (WW), backfat (BF), total number of piglets born (TNB), and number of piglets born alive (NBA) in Landrace pigs. Add, Mat, PE, Gen, Epi, Diff, ***, NS = direct additive genetic solution, maternal genetic solution, permanent environment solution, genetic model, epigenetic model, difference between genetic and epigenetic solutions, significant at 0.1% by a paired *t*-test, and not significant (*P* > 0.05), respectively.

A paired *t*-test at 5% significance level was applied to evaluate the mean difference among the solutions from the BLUP and Epi-BLUP models for the effects mentioned above and are presented in Figure 3. The mean difference between the direct additive and maternal genetic solutions between the BLUP and Epi-BLUP models for all traits were statistically significant. No statistical significance was observed for the mean difference between the permanent environmental solutions comparing the results from the BLUP and Epi-BLUP models.

**FIGURE 4:**
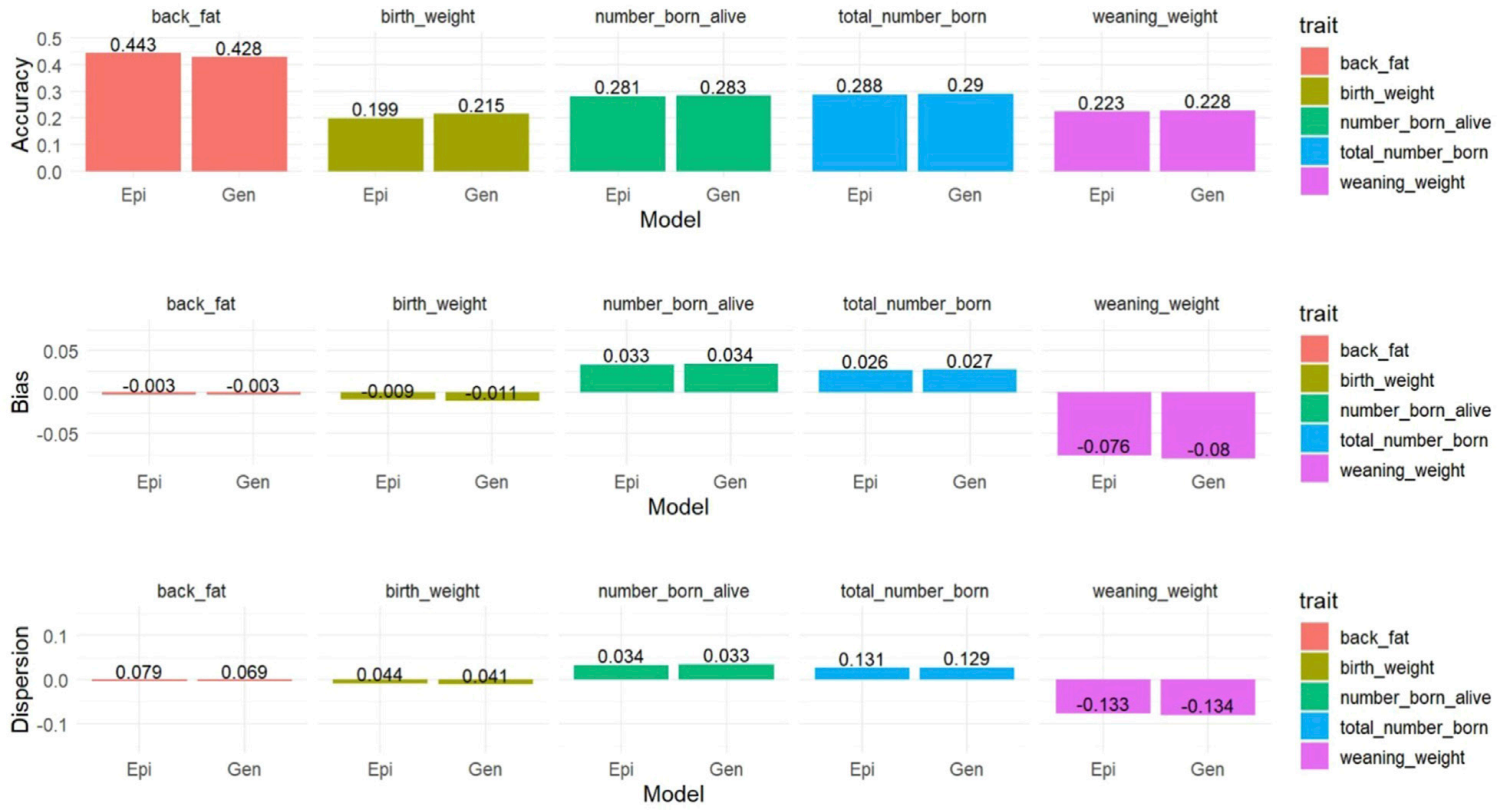
Prediction accuracy, bias, and dispersion of the estimated breeding values of focal animals for birth weight (birth_weight), weaning weight (weaning_weight), backfat (back_fat), total number of piglets born (total_number_born), and number of piglets born alive (number_born_alive) in Landrace pigs. Epi, Gen, = epigenetic model and genetic model, respectively.

Only the genetic and best epigenetic models were used for comparing the prediction results using the LR method. Prediction accuracies ranged between 0.199 and 0.443 for the Epi-BLUP with λ = 0.05 in the BW and Epi-BLUP with λ = 0.10 in the BF, respectively (Figure 4). Prediction bias ranged between −0.080 and 0.034 for the BLUP model on WW and NBA, respectively. The dispersion ranged between −0.134 and 0.131 for the WW BLUP and TNB Epi-BLUP with λ = 0.05, respectively. No substantial differences were observed between the BLUP and Epi-BLUP models for the traits evaluated based on all predictivity measures (accuracy, bias, and dispersion), indicating that the inclusion of transgenerational epigenetic effects did not affect the prediction of the breeding values in the focal animals.

## 4 Discussion

Epigenetics is an important source of phenotypic variation in complex traits, which can also substantially contribute to evolution in livestock (David and Ricard, 2019). Epigenetic changes can be environmentally induced, and then inherited and cause changes in phenotypic expression (Tal et al., 2010). There is limited knowledge about the proportion of the phenotypic variation explained by epigenetic marks in livestock. In this study, we present estimates of transgenerational epigenetic variance and heritability for complex traits in pigs based on routinely recorded datasets and quantitative methods. We also discuss the challenges of conducting such analyses and directions for future studies.

### 4.1 Model comparison

Epigenetic models are quite complex. Even though there is the assumption that there is independence among genetic, epigenetic, and residuals (Tal et al., 2010; Varona et al., 2015), non-negligible linear association between those components exists, as shown in Figure 1. Varona et al. (2015) also reported a posterior association between the transgenerational epigenetic and the additive genetic and residual variance components. Even though there were previous studies that used REML-based methods to estimate transgenerational epigenetic variances (Paiva et al., 2018a; Paiva et al., 2018b), Bayesian inference was preferred to conduct the analysis in this study. Among the main properties of Bayesian inference, it can be highlighted the fact that the variance components are never out of parameter space (Box and Tiao, 1973), and inferences are exact for any sample size (Gianola and Fernando, 1986). We also tried to fit AI-REML-based epigenetic models, but the results were not stable. The number or iterations to converge was very large for some models/traits using AI-REML and many epigenetic models failed to converge (Supplementary Material S3), which is expected as REML estimation is sensitive to complex models (Gianola and Fernando, 1986; Misztal, 2008). The advantages of Bayesian- over REML-based methods make the former more robust for complicated models than the latter. In this sense, only Bayesian inference results were kept in the main text.

All epigenetic models, including or not genomic information, using Bayesian inference converged (Table 2) given the criteria used and led to similar best λ values across traits (0.05 for all traits, except BF, which was 0.10). Epigenetic reprogramming can be produced in laboratory in many ways (Cantone and Fisher, 2013; Basu and Tiwari, 2021), but naturally happens twice during mammalian life (gametogenesis and preimplantation development) (Lacal and Ventura, 2018). In the context of this study, we expect that epigenetic reprogramming is less likely to be influenced by traits such as litter size or backfat depth in sows; rather, it is more likely to influence these traits. Among the factors that affect epigenetic reprograming, there are reports of sex (Lacal and Ventura, 2018), age (Caulton et al., 2022), and environmental factors/stress (Tal et al., 2010).

Even though the Epi-ssGBLUP provided lower DIC, the Epi-BLUP models were chosen as the best because of the clear inflation in the additive genetic, hence phenotypic, variances in the epigenetic models including genomic information. Large changes in variance components previously estimated by introducing new random effects indicate non-orthogonality of the effects in the model (Vitezica et al., 2017), which is related to not accounting for significant interaction between random effects, i.e., their covariance matrices (Zhou et al., 2020). This was not observed in the pedigree-based epigenetic models for all traits (Tables 3–7), i.e., no substantial changes were observed in the variance components when adding the transgenerational epigenetic effect to the genetic model (moving from the BLUP to the Epi-BLUP model). Orthogonality is a very important and useful property because it directly affects the partition of the genetic effects (Vitezica et al., 2017), disqualifying epigenetic models including genomic information (Epi-ssGBLUP) for variance component estimation. Given the current derivation of the epigenetic relationship matrix (Tal et al., 2010; Varona et al., 2015) and the analysis framework employed, i.e., independence of genetic and epigenetic effects, the Epi-BLUP models are more reliable than Epi-ssGBLUP. As the variance components and genetic parameters using a genetic or genomic model (BLUP and ssGBLUP, respectively) were not substantially different, the BLUP model was used as the benchmark for comparisons with Epi-BLUP models because they use the same additive genetic relationship matrix. Similarities between the variance components and genetic parameters estimated using pedigree and genomic models were also reported in the literature by Aldridge et al. (2020), which recommended the use of the pedigree relationship matrix for variance component estimation due to easier implementation.

Modeling the covariance between epigenetic and other random effects in the model could be beneficial, especially for the Epi-ssGBLUP, where the non-orthogonality was evident. To model the interaction between additive genetic effects based on genomics and transcriptomic data, Perez et al. (2022) used Hadamard products between the covariance matrices of these effects and, as an alternative, developed a model where these effects were independent. The independence between the correlated genomic and transcriptomic information was achieved by decorrelating these effects using a “smoother matrix,” which relied on the assumption that the transcriptomic was conditioned to the genomic information, which was called the GTCBLUP model (Perez et al., 2022). Using an interaction effect in the model resulted in non-stable estimates of the variance components, while the GTCBLUP model resulted in a stable partition of the variance components (Perez et al., 2022). Since epigenetic variance originates from epigenetic marks themselves, applying a model similar to that of Perez et al. (2022) would require incorporating epigenomic information (e.g., DNA methylation) into the animal model and conducting the appropriate statistical analysis. Unfortunately, no epigenomic information was present for the animals included in the study. Zhou et al. (2020) proposed a model that considers correlations between random effects in genetic models and showed the importance of modeling the covariance between these effects when non-negligible associations exist (not always linear associations). The core of the work of Zhou et al. (2020) was in providing a framework for multiomics data analyses, but it can be applied in the future for fitting epigenetic models including genomic information. This is because, in the end, the problem of fitting these models is the same: similarity or dissimilarity between the covariance matrices associated with the random effects in the model. However, more theoretical work to understand the association between transgenerational epigenetic and genomic information is required to make assumptions about how to model these effects and their interactions, which were beyond the scope of the present work.

Epigenetics affects the phenotypic variation through changes in gene expression (Heard and Martienssen, 2014), which means that even though the epigenome is not expected to change the DNA sequence, it is modulating or interacting with it during phenotypic expression. In the same way, as epigenetic changes can be stress- or environmentally induced (Ibeagha-Awemu and Yu, 2013), non-negligible associations between epigenetic and residual effects could be produced. Just assuming independence between epigenetic and other effects affecting the phenotypic variation resulted in simpler mathematical work in deriving the covariance between relatives from a quantitative genetics perspective. Even though not optimal from a biological point of view, the assumptions originally made can be considered robust enough in epigenetic models not including genomic information as these models met desired expectations, e.g., orthogonality of the effects. The problems related to non-orthogonality between the additive genetic and epigenetic effects in the genomic analysis (between H and Λ instead of between A and Λ ) may be related to the fact that the epigenetic relationships were derived based on the pedigree-based average relationships. Genomic information provides realized genomic relationships at the QTL level, which changes the additive genetic relationship matrix by adjusting the average relationships to observed values and propagating this information to non-genotyped related to genotyped individuals (Legarra et al., 2009). Changes in the average relationships in closely related individuals plus adding relationship coefficients to animals without pedigree links in the H matrix may have broken important assumptions to hold non-orthogonality when genomic information was added to the epigenic models.

Assuming 0.05 to 0.10 and the best λ values (range of best λ across traits – Table 2), 10%–20% of the epigenetic markers are being inherited in the studied population, i.e., 80%–90% of the epigenetic marks are being reset. No previous reports for the λ , reset, or percentage of epigenetic marks inherited in pigs were found in the literature. The expectation is that the percentage of epigenetic marks being inherited should not be high because it results from an error in resetting the epigenetic marks from one generation to the next (Tal et al., 2010; Basu and Tiwari, 2021), which agrees with our results. Nevertheless, new studies including epigenetic marks (DNA methylations, chromatin remodeling, miRNA, among others) at the individual level could provide insights into the estimation of the reset and inheritance of epigenetic marks.

### 4.2 Variance components and genetic parameters

The variance components and genetic parameters from the BLUP and ssGBLUP models were consistent with the values observed in the literature for all traits evaluated. Alves et al. (2018) reported heritability estimates of 0.05 and 0.07 for the direct additive genetic effects in BW of Canadian Landrace pigs with two datasets. The maternal genetic heritability for the BW presented by Alves et al. (2018), 0.27, was higher than the one observed in this study (∼0.13 for the BLUP, ssGBLUP, and Epi-BLUP models – Table 3), while the proportion of the phenotypic variation explained by the common litter effect was lower (0.17, compared to ∼0.30 from the BLUP, ssGBLUP, and Epi-BLUP models). Even though the base genetic models were the same (fixed effects plus direct and maternal genetic additive and common litter effects), the population and data structure were different (e.g., different numbers of individuals with phenotypes and the pedigree, sires, and dams, between others), which can explain differences in some of the genetic parameters. In general, according to Zaalberg et al. (2023) based on a range of literature reports (Roehe, 1999; Kaufmann et al., 2000; Grandinson et al., 2002), the range of values for the direct and maternal genetic heritability for BW in conventional maternal pig breeds (Landrace and Yorkshire) is between 0.04 to 0.15 and 0.14 to 0.28, respectively. The negative correlation between the direct additive and maternal genetic effects observed was also reported in previous studies (Zhang et al., 2000; Grandinson et al., 2002; Arango et al., 2006; Muns et al., 2013; Alves et al., 2018). This can be explained by the fact that the data structure might not be optimal to properly estimate the correlation between the direct additive and maternal genetic effects or because non-negligible cross-fostering exists (Alves et al., 2018). Cross-fostering information was not known in this study.

Birth weight is an important trait in the swine industry because it is related to piglet survival, behavior, and weight gain (Ayuso et al., 2020; Knol et al., 2022). Low BW is associated with poor gastrointestinal development, which impacts nutrient digestion, absorption, and transportation, implying future health, welfare, and market problems in pigs (Ayuso et al., 2020). Considering the proportion of phenotypic variance explained by the epigenetic effect in the piglet’s BW (0.33 – Table 3), attention should be given to the sow environment during pregnancy, such as weather, sanitary management, and nutrition, as these components can induce epigenetic changes in the progeny (Ibeagha-Awemu and Yu, 2021).

The heritability estimates for the direct genetic effect of the WW in pigs range from 0.01 to 0.22 (Tomiyama et al., 2010; Muns et al., 2013; Dufrasne et al., 2014; Jiao et al., 2014; Alves et al., 2018; Zaalberg et al., 2023) and for the maternal genetic effect from 0.06 to 0.24 (Grandinson et al., 2002; Tomiyama et al., 2010; Jiao et al., 2014; Alves et al., 2018). Similar to previous studies listed above, the heritability of the maternal genetic component reduced from birth to weaning, which reflects the decrease in contribution of the sow effect to the individual body weight of her offspring. The individual’s own genetics is expected to be more important as the pigs age (Alves et al., 2018; Muns et al., 2013). Differences in the heritability estimates for WW among studies, beyond the previous causes mentioned before (i.e., data structure and population), could also be due to the model used. The correlation between the direct additive and maternal genetic effects was not significant in this study for WW (Table 4), as reported in other studies (Alves et al., 2018; Kaufmann et al., 2000; Grandinson et al., 2002). Data structure and cross-fostering can also be reasons for the lack of significance in the correlation between the direct additive and maternal genetic effects for WW.

The weaning process is a stressful situation for both piglets and dams, marked by the time when the piglet leaves the maternal to a different environment and changes the diet, characterizing social and dietary stress (Corbett et al., 2013; Ibeagha-Awemu and Yu, 2021). The WW can be used to assess the success of the lactation phase, and it is the start of the period when the pigs will express their genetic potential without their mother’s direct contributions. Heavy pigs at weaning tend to also be heavy at birth and slaughter age and have faster growth rates, achieving the age to slaughter earlier than lighter pigs (Wolter and Ellis, 2001). Transgenerational epigenetic heritability for WW in Landrace pigs explained more than 30% of the phenotypic variation, and more research is needed to understand the impact of epigenetics due to production management practices. This way, strategies to minimize stress at weaning are important for reducing the potential negative impacts on the pig’s performance.

Beyond growth, desirable body composition characteristics are important breeding goals in the pig industry. The genetic parameters for BF in this study are within the range reported in the literature for body composition traits (0.33–0.65), which are known to have moderate to high direct narrow-sense heritability (Bryner et al., 1992; Singh et al., 2001; Kim et al., 2004; Akanno et al., 2013; Khanal et al., 2019; Willson et al., 2020). Maternal genetic heritability was also a significant variance component in a genetic model for BF in pigs in a previous study (Bryner et al., 1992), with the estimate higher than the one observed in this study (0.11). Bryner et al. (1992) studied Yorkshire pigs and found a negative genetic correlation between direct and maternal genetic components for BF, which was stronger than the estimate observed in this study (−0.51). Negative correlations between direct and maternal genetic effects for BF can happen due to negative genetic association between these effects or data structure and modeling (Bryner et al. ,1992). These results suggest that BF can have a high response to direct selection and that maternal effects should be considered in the genetic models of the studied population.

Beyond the high narrow-sense heritability, BF can be an indicator of the overall body fat and be easily measured through ultrasound, being an important criterion to select pigs for lean carcass (Gozalo-Marcilla et al., 2021). The reduction in carcass fatness can imply higher growth efficiency and lean meat content (Lonergan et al., 2001). In addition, BF is important for the reproductive performance of sows (Roongsitthichai and Tummaruk, 2014). Roongsitthichai and Tummaruk (2014) reviewed the importance of BF for reproductive traits in pigs and reported that gilts with higher BF attained puberty earlier, delivered one more pig, produced heavier piglets with higher growth rates, and had shorter weaning to service intervals. Interestingly, the transgenerational epigenetic heritability for BF also explained more than 30% of the phenotypic variation, similar to BW and WW. Considering the importance of BF for the pig industry and the percentage of phenotypic variance explained by the transgenerational epigenetic effects, more research on this topic is needed.

The variance components and genetic parameters for TNB and NBA were similar in this research and, therefore, they were discussed together. This similarity in the genetic parameters for TNB and NBA was reported before (Akanno et al., 2013; Putz et al., 2015; Ogawa et al., 2019; Paixao et al., 2019; Yang et al., 2023) with both traits being highly genetically correlated (Alves et al., 2018). Reproductive traits usually present low heritability and the range of heritability estimates for TNB and NBA is from 0.01 up to 0.20 (Akanno et al., 2013; Putz et al., 2015; Alves et al., 2018; Ogawa et al., 2019; Paixao et al., 2019; Yang et al., 2023), with most of the estimates varying from 0.06 to 0.12. The repeatability of the TNB and NBA estimates in the literature range from 0.03 to 0.24 (Alves et al., 2018; Ogawa et al., 2019; Paixao et al., 2019; Yang et al., 2023). The heritability and repeatability estimates for TNB and NBA in this study are close to the estimates from previous studies. As previously discussed, the population and data structure are the main reasons for the differences observed in the literature, as the models for these traits tend to be similar.

As expected, and different from what was observed for growth and body composition traits, the transgenerational epigenetic variance and heritability were low for TNB and NBA. We expected that traits measured late in life would be less impacted by epigenetic effects because: 1) the direct genetic effects tend to increase in importance as the animals age; and 2) temporary environmental effects (environmental effects not fully captured by data structure or model) could be the main source of environmental variation. At this point, it is important to remember that transgenerational epigenetic effects are due to epigenetic markers inherited, and they should affect the animal’s life permanently (Heard and Martienssen, 2014). In this context, the source of transgenerational epigenetic variance in the repeatability models should also be considered. Different from single-measurement models in this study, as for BW, WW, and BF, the transgenerational epigenetic variance in the repeatability models for the TNB and NBA were all extracted from the permanent environment effect. The permanent environment accounts for non-additive genetic effects (i.e., dominance, epistasis), as shown by Vitezica et al. (2018), and any other effect with a long-lasting impact on the animal’s life. With this result, it is possible to conclude that the transgenerational epigenetic effect could be treated as a non-additive genetic effect in repeatability models and being accounted for in the genetic models through multiple measurements. This way, it becomes easy to see that including the transgenerational epigenetic effects in the genetic models is more important for traits that can be measured only once in the animal’s life, e.g., BW, WW, and age at first farrowing.

Beyond the repeatability models, another alternative to fit permanent environment effects in a genetic model is by using random regression models (RRM), which also allows for modeling the phenotype (and its components) trajectory through covariance functions (Schaeffer, 2004). In this context, we also expect that RRM can account for the transgenerational epigenetic effects in the permanent environment effects, but no precedents were found in the literature. We recommend such studies with better data structures (e.g., multiple measurements for growth traits early in life) to test this hypothesis in the future. However, the confounding between the transgenerational epigenetic and permanent environment effects and the impacts of this in convergence should be considered, especially for complex models like RRM.

Several differentially methylated regions (DMRs) were reported in the literature for BW (Ayuso et al., 2020), WW (Corbett et al., 2013), BF (Li et al., 2012; Zhang et al., 2016; Wang K. et al., 2022), and TNB and NBA (Hwang et al., 2017; An et al., 2019) in pigs. The DMRs are the most studied epigenetic marks and affect gene expression (Ayuso et al., 2020; Corbett et al., 2013; Li et al., 2012; Zhang et al., 2016; Wang K. et al., 2022; Hwang et al., 2017; An et al., 2019), consequently, can affect the phenotypic variation and be the cause of the epigenetic heritability estimated for the traits investigated in this study. In previous reports, the transgenerational epigenetic heritability was 0.04 for BW in the Pirenaica beef cattle (Varona et al., 2015), and 0.00 to 0.10 for body weight traits in meat quails (Paiva et al., 2018b), and 0.00 to 0.07 in egg traits in meat quails (Paiva et al., 2018a). No previous reports on pigs were found. The species, models, and production systems, among other factors previously mentioned, may be the cause of the difference between the transgenerational epigenetic heritability estimates in the literature and in this study. A reason for different epigenetic heritability across traits could be because epigenetic marks can be linked to QTL with different patterns of linkage disequilibrium (LD) with the traits evaluated. In other words, the epigenetic marks can overlap with or be nearby QTL that are in more LD with a specific trait than others. Therefore, epigenetic heritability is expected to be higher for traits that have more QTL affected by epigenetic marks. One way to find insights into this subject would be by crossing DNA methylation maps with QTL maps for traits with known epigenetic heritability, which was not possible in this study because no whole-genome DNA methylation data was available for the animals used. Future work on this topic is recommended. Following Animal QTLdb, which is a public QTL information repository for several farm species, including pigs (Hu et al., 2022), the development of public DNA methylation maps in pigs would also help to integrate information and understand the causes of the transgenerational epigenetic variance and heritability found in this and future studies. DNA methylation maps in pigs are still scarce (Wang and Kadarmideen, 2019).

### 4.3 Prediction results

One of the main objectives of this study was to compare the prediction results from the best epigenetic model against the genetic or genomic model. As there was no stability in the variance components of the Epi-ssGBLUP, shown by the absence of orthogonality in the models, no decorrelations of the relationships were performed, and no predictions were made for Epi-ssGBLUP. The Epi-ssGBLUP may have been affected by an un-modeled covariance among the epigenetic effects and that of the genetic and residual effects, resulting in drastic changes in the variance components. There were no substantial differences between the variance components from the BLUP and ssGBLUP, suggesting that the variance components from the BLUP model are consistent even though the population evaluated is being genomically selected. Using variance components from Epi-BLUP models, which were stable and more reliable, predictions with the Epi-ssGBLUP model were not performed because making predictions in a model using a relationship matrix different from the one used to estimate the variance components is not recommended (Nilforooshan and Ruíz-Flores, 2022). In the end, the BLUP models were used to compare the predictions against the Epi-BLUP models for similar reasons explained in the *Variance Component Estimation* section.

By using all animals present in the variance components estimation analysis, no differences are expected in the genetic evaluation, as the correlation between the EBVs estimated in genetic and epigenetic models was high (>0.85; Figure 2). The same could be interpreted for the other random effects in the models, except for the maternal solutions in the BF (−0.22). It is important to highlight that the correlations presented in Figure 2 should not be interpreted as prediction accuracy. The solutions used to compute the Pearson correlations included all phenotypes available, and all solutions were used only to understand the association between the solutions. A formal comparison of EBV prediction, e.g., prediction accuracies, for selection candidates is provided by the results of the LR method (discussed later). Even though the association between solutions was high, the changes in the means of the solutions were significant, showing a different aggregate response for the solutions, except for the permanent environment. Observing that the epigenetic models had a better fit than the genetic models (please see the *Model comparison* section in the results and discussion), the averages of the solutions for the additive effects of the BW, WW, and BF from the genetic models were underestimated, and the solutions are more dispersed. For the case of the additive solutions for the TNB and NBA and maternal genetic solutions for BW, WW, and BF, the averages from the genetic models are overestimated and more dispersed compared to the epigenetic models. In this case, including the transgenerational epigenetic effects in the genetic models could help properly estimate the average and distribution of the solutions of the mixed model equations, provided that the epigenetic models are better than the genetic models.

The prediction (population) accuracy in pigs usually ranges between 0.05 and 0.82 (reproductive and body composition traits, respectively) (Forni et al., 2011; Hidalgo et al., 2015a; Hidalgo et al., 2015b; Hidalgo et al., 2016; Badke et al., 2014; Fangmann et al., 2017; Oh et al., 2017; Tan et al., 2017; Lee et al., 2020; Zhao et al., 2019; Ardestani et al., 2021; Kjetså et al., 2022; Wang X. et al., 2022; Zhuang et al., 2023). Several factors can affect the prediction accuracies, such as heritability, LD (Meuwissen et al., 2001), population structure, effective population size (Daetwyler et al., 2012), and size of the reference population (Daetwyler et al., 2010). Beyond these factors, the genetic model, accuracy formula used, validation strategy, breed, and the use of commercial crossbreed data also differ between studies and can be a source variation for the prediction accuracy in the pig literature. Using genomic data, Jiao et al. (2014) reported prediction accuracies of 0.194 for BW in Duroc pigs using the BayesA model. Also, using genomic data, Lee et al. (2020) reported prediction accuracies for BW in Yorkshire pigs with the BayesB and BayesC models using panels of different platforms ranging from 0.150 to 0.261. Even though we used only phenotypes and pedigree, the prediction accuracies obtained for the BW were within this range.

A low prediction accuracy (0.098) was reported for WW in Duroc pigs (Jiao et al., 2014), lower than the estimate obtained in this study (∼0.22). The previous authors used the formula from Legarra et al. (2008) to calculate the prediction accuracy and an imputed 60K from a low-density marker panel to perform the genomic predictions. The accuracy formula from Legarra et al. (2008) depends on the correlation between the observed and expected performance divided by the square root of the heritability of the trait, which can be reasonable if there is no selection, and the fixed effects and variance components are properly estimated (Legarra and Reverter, 2018). If there is selection, the accuracy measured by the method proposed by Legarra et al. (2008) underestimates the prediction accuracy, and problems may also arise if the fixed effects and/or variance components are not well estimated (Legarra and Reverter, 2018). In addition, prediction accuracy when using imputed data is also a function of the imputation accuracy, with higher accuracies expected if the imputation accuracy is high (Bolormaa et al., 2015).

Prediction accuracies ranging from 0.285 to 0.527 were observed for BF in Canadian Landrace by using parent average (PA) and several genomic models (Ardestani et al., 2021), in which the lowest prediction accuracy was obtained by PA and the highest by the ssGBLUP model. Ardestani et al. (2021) measured the prediction accuracy as the correlation between (G)EBV and deregressed EBVs. Despite the differences in the accuracy formula and models between our study and Ardestani et al. (2021), the ranges of prediction accuracies were similar. The prediction accuracy for the EBVs of BF in our study was lower than the ones obtained by the genomic models used by Ardestani et al. (2021), but higher than the PA. Jiao et al. (2014) reported a prediction accuracy lower than the one obtained in our study (0.365) using genomic models.

Most studies investigating prediction accuracy in pigs were found in reproductive traits, especially TNB. For NBA, Fangmann et al. (2017) found prediction accuracies ranging from 0.08 to 0.52 using different validation scenarios and accuracy measures in Landrace pigs. In Chinese Yorkshire pigs, Song et al. (2019) reported accuracies ranging from −0.008 to 0.668 for NBA using different SNP panels and validation scenarios. Wang X. et al. (2022) observed prediction accuracies ranging between 0.207 and 0.328 for NBA also in Chinese Yorkshire but based on several machine learning models and genomic BLUP and ssGBLUP. However, it should be noted that the machine learning models may not result in only the predictions of breeding values. The range of accuracy estimates for TNB reported in the literature range from −0.001 to 0.790 (Forni et al., 2011; Hidalgo et al., 2015a; Hidalgo et al., 2015b; Hidalgo et al., 2016; Oh et al., 2017; Song et al., 2019; Kjetså et al., 2022; Wang X. et al., 2022), based on different models, validation strategies, breeds, and other factors as previously mentioned.

In general, prediction accuracy results in pig literature are reported mainly for genomic models, with some studies presenting pedigree-based prediction accuracies and formal comparisons (Forni et al., 2011; Christensen et al., 2012; Tusell et al., 2013; Hidalgo et al., 2015b; Oh et al., 2017; Ardestani et al., 2021). Including genomics in the prediction models tends to increase prediction accuracies because it allows to capture more information to be used to differentiate animals and correct expected relationships (Mendelian sampling) (Georges et al., 2019). Similar or higher prediction accuracies were observed by some authors using only pedigree when compared to genomic relationships (Hidalgo et al., 2015b; Araujo et al., 2023). This may happen when the heritability of the trait is high, pedigree is relatively deep and accurate, there are rare variants with big effects or insufficient genomic information.

Prediction bias calculated as the difference between the (G)EBV estimated in the whole and partial data (Legarra and Reverter, 2018) are scarce in the pig literature. Jang et al. (2023) observed the prediction bias ranging from −0.003 to 0.31 for BF in three terminal pig lines based on different scenarios using preselected variants from whole genome sequence data. The lowest value observed in the BF prediction bias by these authors was the same as the one observed in this study for both BLUP and Epi-BLUP models. Even though only pedigree and phenotypes were used to investigate prediction results in this study, the prediction bias obtained can be considered low, as it ranged between −0.011 and 0.08 (BLUP models for BW and WW, respectively). In the case of the prediction dispersion, which sometimes is called bias, it has been reported as the slope of the regression coefficient of the adjusted phenotype/EBV on the predicted EBV, different from this research that used a deviation from 1. The prediction dispersion (measured as the slope of the regression) in the pig literature ranges between −1.14 and 5.00 (Fangmann et al., 2017; Hidalgo et al., 2015b; Hidalgo et al., 2016; Wang X. et al., 2022), in which the factors that justify the changes in the literature results are similar to those previously stated for the prediction accuracy. The expected/desired value for the dispersion is 1 if measured as the slope of the regression coefficient of the adjusted phenotype/EBV on the predicted EBV or 0 if measured as the deviation from 1 (as performed in this research). Based in the literature cited above, TNB is the trait that presents worse results for dispersion, and it was the trait with the second worse results for dispersion in our study.

To the best of our knowledge, no prediction results for the measures investigated in this research (accuracy, bias, and dispersion of EBV) in genetic models incorporating epigenetic effects have been reported in the literature. This study provides such results for the first time. The similarity between the prediction accuracies, bias, and dispersion in the BLUP and Epi-BLUP models is expected as the transgenerational epigenetic in the Epi-BLUP model is fitted independent of the other random effects, and the orthogonality was present. This shows that including the epigenetic effects in the genetic model does not change the breeding value prediction of focal animals, which suggests that including epigenetic effects in the model is not required from a practical point of view in the current framework (e.g., not including genomic or epigenomic information, not accounting potential interactions). However, it should be noted that the prediction results in this research were not meant to perform model comparison/selection, as this was the purpose of the *Model Comparison* section, in which the DIC was used as a criterion and showed that epigenetic models have the best fit for all traits. Prediction results were included to show potential advantages in breeding values predicted by epigenetic models over regular animal models, which was not the case probably because of the reasons explained above. Nevertheless, more research is needed on this topic. In addition, using epigenetic genetic effects may still be important for performance/phenotype prediction as it contributes to phenotypic variation.

There is significant transgenerational epigenetic variance for growth, body composition, and reproductive traits in Landrace pigs. Theoretical and analytical work should be done to propose methods for deriving a covariance between epigenetic and additive and residual effects, especially for genomic models where there was non-orthogonality of the effects included. The estimated percentage of epigenetic marks being inherited in Landrace pigs ranged from 10% to 20%, while the reset coefficient was between 0.80 and 0.90, considering the traits evaluated. Transgenerational epigenetic heritability (fraction of the phenotypic variance explained by the transgenerational epigenetic effects) is high for growth and body composition traits (>0.30) and low for reproductive traits (∼0.04) in Landrace pigs. The permanent environment effect can capture the transgenerational epigenetic variances, classifying the epigenetic effect as another non-additive genetic effect affecting phenotypic variation and indicating that it is more important to fit epigenetic effects in single-record traits. EBVs from genetic and epigenetic models are highly correlated at the population level, while statistical differences between the mean values exist. Including epigenetic effects in genetic models does not impact the prediction accuracy, bias, and dispersion of the EBVs of focal individuals (young non-phenotyped selection candidates). This work provides a comprehensive investigation of the impact of epigenetic effects on genetic and genomic models from a quantitative point of view, providing a solid base for future studies in this area.

## Data Availability

The data analyzed in this study is subject to the following licenses/restrictions: The phenotypic, pedigree and genomic data used in this study are the property of the industry partner that contributed to the study and therefore are not readily available due to its commercial sensitivity. Requests to access the datasets should be directed to the Smithfield Premium Genetics (SPG). The computing pipelines used in this research are available by request to the corresponding author. Requests to access these datasets should be directed to Yijian Huang, yhuang@smithfield.com.
