# Supplementary material for: Transgenerational epigenetic heritability for growth, body composition, and reproductive traits in Landrace pigs
Source: Front Genet. 2025 Jan 23;15:1526473. doi: 10.3389/fgene.2024.1526473 (PMC11799271; doi:10.3389/fgene.2024.1526473)
Supplement: Supplementary file 1 [file Table1.docx]

Supplementary Material 1

# Supplementary Data

Python code to create the upper triangular with diagonal epigenetic relationship matrix in the long format, i.e., with three columns (i, j, and value):

Epinv = open('Epinv_function.py', 'w+')

Epinv.write('''#!/usr/bin/env python3

import pandas as pd

from itertools import product

def getEpinv(ped_file,lambda_value):

ped = pd.read_csv(ped_file, header=None, delimiter=,' ')

ped = ped.drop([3], axis=1)

ped.columns = ['id','sire','dam']

Epinv = pd.DataFrame(list(product(ped['id'], ped['id'])))

Epinv = pd.concat([Epinv,Epinv], axis=1)

Epinv['ij'] = 0

Epinv.columns = ['i','j','i_i','j_i','ij']

Epinv.set_index(['i_i','j_i'], inplace=True)

Epinv['ij'] = pd.to_numeric(Epinv['ij'], downcast='float')

Epinv = Epinv[(Epinv.j >= Epinv.i)]

ID_list = ped['id'].values.tolist()

lambda_value=lambda_value

for ID in ID_list:

ID_i = ped.loc[ped['id'] == ID].reset_index()

SIRE = ID_i.at[0,'sire']

DAM = ID_i.at[0,'dam']

if SIRE == DAM == 0: # step 1: sire and dam unknown

Epinv.at[(ID,ID),'ij'] = Epinv.at[(ID,ID),'ij'] + 1

if (SIRE != 0 and DAM == 0) or (SIRE == 0 and DAM != 0): # step 2: only one parent known

Epinv.at[(ID,ID),'ij'] = Epinv.at[(ID,ID),'ij'] + 1/(1-lambda_value**2)

if SIRE != 0: # if the sire is the known parent

Epinv.at[(SIRE,SIRE),'ij'] = Epinv.at[(SIRE,SIRE),'ij'] + lambda_value**2/(1-lambda_value**2)

Epinv.at[(SIRE,ID),'ij'] = Epinv.at[(SIRE,ID),'ij'] + -lambda_value/(1-lambda_value**2)

if DAM != 0: # if the dam is the known parent

Epinv.at[(DAM,DAM),'ij'] = Epinv.at[(DAM,DAM),'ij'] + lambda_value**2/(1-lambda_value**2)

Epinv.at[(DAM,ID),'ij'] = Epinv.at[(DAM,ID),'ij'] + -lambda_value/(1-lambda_value**2)

if SIRE != 0 and DAM != 0: # step 3: both parents known

Epinv.at[(ID,ID),'ij'] = Epinv.at[(ID,ID),'ij'] + 1/(1-2*lambda_value**2)

Epinv.at[(SIRE,ID),'ij'] = Epinv.at[(SIRE,ID),'ij'] + -lambda_value/(1-2*lambda_value**2)

Epinv.at[(DAM,ID),'ij'] = Epinv.at[(DAM,ID),'ij'] + -lambda_value/(1-2*lambda_value**2)

Epinv.at[(SIRE,SIRE),'ij'] = Epinv.at[(SIRE,SIRE),'ij'] + lambda_value**2/(1-2*lambda_value**2)

Epinv.at[(DAM,DAM),'ij'] = Epinv.at[(DAM,DAM),'ij'] + lambda_value**2/(1-2*lambda_value**2)

if SIRE > DAM:

Epinv.at[(DAM,SIRE),'ij'] = Epinv.at[(DAM,SIRE),'ij'] + lambda_value**2/(1-2*lambda_value**2)

if DAM > SIRE:

Epinv.at[(SIRE,DAM),'ij'] = Epinv.at[(SIRE,DAM),'ij'] + lambda_value**2/(1-2*lambda_value**2)

Epinv = Epinv[(Epinv.ij != 0)]

Epinv.to_csv('Epinv.txt', header=None, index=None, sep=' ', mode='w')

getEpinv(ped_file="renumbered_ped.txt",lambda_value=VALUE_FOR_LAMBDA)

''')

Epinv.close()
