# Supplementary material for: Transgenerational epigenetic heritability for growth, body composition, and reproductive traits in Landrace pigs
Source: Front Genet. 2025 Jan 23;15:1526473. doi: 10.3389/fgene.2024.1526473 (PMC11799271; doi:10.3389/fgene.2024.1526473)
Supplement: Supplementary file 2 [file Table2.docx]

Supplementary Material 2

# Supplementary Data

**Table S1.** Akaike information criteria (AIC) of genetic models including different sets of random effects in the models for birth weight (BW), weaning weight (WW), back fat thickness (BF), total number of piglets born (TNB), and number of piglets born alive (NBA) in Landrace pigs.

| Random effects^a^ | BW | WW | BF | TNB | NBA |
| --- | --- | --- | --- | --- | --- |
| add | 24,032.68 | 54,278.43 | -14,852.13 | ***52,845.10*** | ***51,802.931*** |
| add_c | 23,329.48 | 53,760.17 | -14,899.93 | NC | NC |
| add_mat_cov | -8,966.79 | 21,474.89 | ***-47,565.92*** | NC | NC |
| add_mat_cov_c | ***-9,499.99^b^*** | 21,042.92 | NC | NC | NC |
| add_mat_cov_mp | -9,017.52 | 21,431.30 | NC | NC | NC |
| add_mat_cov_mp_c | NC^3^ | 46,617.70 | NC | NC | NC |
| add_mat_no_cov | -8,957.14 | 21,477.69 | -47,554.60 | NC | NC |
| add_mat_no_cov_c | -9,482.86 | ***21,041.39*** | NC | NC | NC |
| add_mat_no_cov_mp | -8,926.72 | 21,437.17 | NC | NC | NC |
| add_mat_no_cov_mp_c | NC | NC | NC | NC | NC |

^a^add = additive genetic only; add_c = additive genetic + common environment; add_mat_cov = additive genetic + maternal genetic with covariance between additive and maternal genetic effects; add_mat_cov_c = additive genetic + maternal genetic + common environment with covariance between additive and maternal genetic effects; add_mat_cov_mp = additive genetic + maternal genetic + maternal permanent environment effect with covariance between additive and maternal genetic effects; add_mat_cov_mp_c = additive genetic + maternal genetic + maternal permanent environment + common environment effect with covariance between additive and maternal genetic effects; add_mat_no_cov = additive genetic + maternal genetic with NO covariance between additive and maternal genetic effects; add_mat_no_cov_c = additive genetic + maternal genetic + common environment with NO covariance between additive and maternal genetic effects; add_mat_no_cov_mp = additive genetic + maternal genetic + maternal permanent environment effect with NO covariance between additive and maternal genetic effects; add_mat_no_cov_mp_c = additive genetic + maternal genetic + maternal permanent environment + common environment effect with NO covariance between additive and maternal genetic effects. The fixed effects used in the genetic models for each trait were defined as the significant effects (*P* < 0.05) using a linear model from a set of available variables that could potentially affect the traits of interest. Please see the main text for more information regarding the full models for each trait.
