# Supplementary material for: Transgenerational epigenetic heritability for growth, body composition, and reproductive traits in Landrace pigs
Source: Front Genet. 2025 Jan 23;15:1526473. doi: 10.3389/fgene.2024.1526473 (PMC11799271; doi:10.3389/fgene.2024.1526473)
Supplement: Supplementary file 3 [file Table3.docx]

Supplementary Material 3

# Supplementary Data

**Table S1.** Akaike Information Criteria (AIC) of genetic and epigenetic models including or not genomic information in the relationship (REL) matrix for birth weight (BW), weaning weight (WW), backfat thickness (BF), total number of piglets born (TNB), and number of piglets born alive (NBA) in Landrace pigs

| **Model** | **BW** | **WW** | **BF** | **TNB** | **NBA** |
| --- | --- | --- | --- | --- | --- |
| BLUP^a^ | -9499.99 | 21,041.39 | -47,565.92 | 52,845.10 | 51,802.93 |
| Epi-BLUP^b^ |  |  |  |  |  |
| $\lambda$ = 0.05 | -9499.20 | 21,043.40 | -47,564.74 | 52,845.10 | 51,804.94 |
| $\lambda$ = 0.10 | -9499.19 | 21,043.39 | -47,566.42 | 52,845.10 | 51,804.93 |
| $\lambda$ = 0.15 | -9498.89 | 21,043.37 | -47,567.64 | 52,845.10 | 51,804.93 |
| $\lambda$ = 0.20 | *-9517.29^e^* | 21,043.36 | *-47,583.19* | 52,845.10 | 51,804.93 |
| $\lambda$ = 0.25 | -9498.49 | 21,043.37 | -47,569.00 | 52,845.10 | 51,804.93 |
| $\lambda$ = 0.30 | -9498.38 | 21,043.38 | -47,568.78 | 52,845.10 | 51,804.93 |
| $\lambda$ = 0.35 | -9498.31 | 21,043.39 | -47,567.98 | 52,845.10 | 51,804.93 |
| $\lambda$ = 0.40 | -9498.26 | *21,021.91* | -47,566.75 | 52,845.10 | 51,804.93 |
| $\lambda$ = 0.45 | NC^6^ | 21,043.39 | -47,565.38 | 52,845.10 | 51,805.68 |
| ssGBLUP^c^ | *-27,539.69* | *3049.39* | -65,667.24 | *52,795.73* | *51,748.48* |
| Epi-ssGBLUP^d^ |  |  |  |  |  |
| $\lambda$ = 0.05 | NC | 3063.54 | -65,434.23 | 52,831.77 | 51,791.72 |
| $\lambda$ = 0.10 | -27,508.03 | 3063.45 | -65,436.74 | 52,831.77 | 51,791.72 |
| $\lambda$ = 0.15 | -27,507.64 | 3063.37 | -65,460.50 | 52,831.77 | 51,791.72 |
| $\lambda$ = 0.20 | -27,507.39 | 3063.33 | -65,512.92 | 52,831.77 | 51,791.72 |
| $\lambda$ = 0.25 | -27,507.23 | 3063.33 | NC | 52,831.77 | 51,791.72 |
| $\lambda$ = 0.30 | -27,507.14 | 3063.35 | NC | 52,831.77 | 51,791.72 |
| $\lambda$ = 0.35 | -27,507.08 | 3063.37 | NC | 52,831.77 | 51,791.72 |
| $\lambda$ = 0.40 | -27,507.02 | 3063.41 | *-65,538.28* | 52,831.77 | 51,791.72 |
| $\lambda$ = 0.45 | -27,506.96 | 3063.44 | -65,537.27 | 52,831.77 | 51,791.72 |

^a^Best Linear Unbiased Prediction using the **A** matrix as the relationship matrix for the additive genetic effect. ^b^Epigenetic models obtained by including the transgenerational epigenetic effect in the BLUP model with their respective recursive parameter ($\lambda$) values.

^c^Single-step genomic BLUP obtained by replacing the **A** for the **H** (combined pedigree and genomic relationship matrix) matrix.

^d^Epigenetic models including genomic information obtained by expanding the ssGBLUP model by including the transgenerational epigenetic effect with their respectively $\lambda$ values below.

^e^Italic AIC values indicate the lowest values, in which two values were defined per trait (using or not genomic information**)**.

^f^The model did not converge.

The ssGBLUP and Epi-ssGBLUP models presented lower AIC values for all traits compared to the BLUP and Epi-BLUP models (Table 2). However, differently from the BLUP-based models, the Epi-ssGBLUP presented lower AIC value compared to the ssGBLUP only for BF (-65,538.28 and -65,667.24, respectively), in which the $\lambda$ = 0.40 provided the best fit in the Epi-ssGBLUP for BF. Different $\lambda$ values had little to no impact in the Epi-ssGBLUP models for BW, WW, TNB, and NBA. The number of models that did not converge was larger for Epi-ssGBLUP as compared to Epi-BLUP-based models (n = 4 and 1, respectively) considering all traits.

**Table S2.** Variance components and genetic parameters for birth weight of Landrace pigs using genetic and epigenetic models with pedigree- or single-step Genomic BLUP based on AIREML approach.

| Parameter^a^ | Genetic | Epigenetic ($\lambda=0.20$) | Genomic | Epi_geno ($\lambda=0.10$)^b^ |
| --- | --- | --- | --- | --- |
| N iterations | 15 | 16 | 16 | 17 |
| AIC | -9,499.99 | -9,517.29 | -27,539.69 | -27,508.03 |
| $\sigma_{\mathbf{u}}^{2}$ | 0.025 (0.010)^c^ | 0.014 (0.011) | 0.036 (0.016) | 0.069 (0.028) |
| $\sigma_{\mathbf{m}}^{2}$ | 0.071 (0.013) | 0.069 (0.013) | 0.072 (0.012) | 0.248 (0.043) |
| $\sigma_{\mathbf{u,m}}$ | -0.004 (0.011) | -0.001 (0.011) | -0.007 (0.010) | 0.002 (0.037) |
| $\sigma_{\mathbf{q}}^{2}$ | 0.167 (0.009) | 0.166 (0.009) | 0.167 (0.009) | 0.155 (0.009) |
| $\sigma_{\boldsymbol{\xi}}^{2}$ | - | 0.075 (0.090) | - | 0.187 (0.164) |
| $\sigma_{\mathbf{e}}^{2}$ | 0.302 (0.008) | 0.239 (0.078) | 0.296 (0.009) | 0.096 (0.156) |
| $\sigma_{P}^{2}$ | 0.561 (0.009) | 0.560 (0.009) | 0.564 (0.010) | 0.757 (0.027) |
| $h_{\mathbf{u}}^{2}$ | 0.044 (0.019) | 0.024 (0.028) | 0.065 (0.020) | 0.091 (0.036) |
| $h_{\mathbf{m}}^{2}$ | 0.127 (0.022) | 0.123 (0.023) | 0.127 (0.020) | 0.327 (0.052) |
| $r_{\mathbf{u}\boldsymbol{,}\mathbf{m}}$ | -0.098 (0.416) | -0.037 (0.522) | -0.130 (0.197) | 0.015 (0.373) |
| $q^{2}$ | 0.298 (0.015) | 0.296 (0.015) | 0.296 (0.015) | 0.205 (0.015) |
| $h_{\boldsymbol{\xi}}^{2}$ | - | 0.133 (0.162) | - | 0.247 (0.220) |

^a^N iterations = number of iterations needed to reach convergence; AIC = Akaike Information Criteria; $\sigma_{\mathbf{u}}^{2}$, $\sigma_{\mathbf{m}}^{2}$, $\sigma_{\mathbf{u,m}}$, $\sigma_{\mathbf{q}}^{2}$, $\sigma_{\boldsymbol{\xi}}^{2}$, $\sigma_{\mathbf{e}}^{2}$, $h_{\mathbf{u}}^{2}$, $h_{\mathbf{m}}^{2}$, $r_{\mathbf{u}\boldsymbol{,}\mathbf{m}}$, $q^{2}$, and $h_{\boldsymbol{\xi}}^{2}$ are the additive genetic, maternal genetic, covariance between additive and maternal genetic, common environment, transgenerational epigenetic, residual, and phenotypic variances, and additive genetic heritability, maternal genetic heritability, correlation between additive and maternal genetic effects, fraction of the phenotypic variance explained by the common environment effect, and transgenerational epigenetic heritability, respectively. ^b^Epi_genomic was used to emphasize that it is an epigenetic model including genomic information (no epigenomic information, e.g., DNA methylation) was included. ^s^Standard error are presented within parenthesis.

**Table S3.** Variance components and genetic parameters for the weaning weight of Landrace pigs using genetic and epigenetic models with pedigree- or single-step genomic BLUP based on the AIREML method.

| Parameter^a^ | Genetic | Epigenetic ($\lambda=0.40$) | Genomic | Epi_geno ($\lambda=0.25$)^b^ |
| --- | --- | --- | --- | --- |
| N iterations | 16 | 9217 | 16 | 17 |
| AIC | 21,041.387 | 21,021.914 | 3,049.387 | 3,063.329 |
| $\sigma_{\mathbf{u}}^{2}$ | 0.349 (0.149) | 0.345 (0.119) | 0.374 (0.132) | 1.115 (0.505) |
| $\sigma_{\mathbf{m}}^{2}$ | 0.354 (0.102) | 0.354 (0.090) | 0.265 (0.080) | 1.171 (0.320) |
| $\sigma_{\mathbf{q}}^{2}$ | 2.591 (0.146) | 2.591 (0.144) | 2.651 (0.140) | 2.553 (0.150) |
| $\sigma_{\boldsymbol{\xi}}^{2}$ | - | 0.007 (0.004) | - | 0.321 (0.720) |
| $\sigma_{\mathbf{e}}^{2}$ | 5.767 (0.142) | 5.764 (0.133) | 5.756 (0.136) | 5.093 (0.541) |
| $\sigma_{P}^{2}$ | 9.061 (0.137) | 9.061 (0.136) | 9.045 (0.136) | 10.253 (0.323) |
| $h_{\mathbf{u}}^{2}$ | 0.039 (0.016) | 0.038 (0.013) | 0.041 (0.015) | 0.109 (0.048) |
| $h_{\mathbf{m}}^{2}$ | 0.039 (0.011) | 0.039 (0.010) | 0.029 (0.009) | 0.114 (0.029) |
| $q^{2}$ | 0.286 (0.015) | 0.286 (0.014) | 0.293 (0.014) | 0.249 (0.017) |
| $h_{\boldsymbol{\xi}}^{2}$ | - | 0.001 (0.000) | - | 0.031 (0.071) |

^a^N iterations = number of iterations to converge; AIC = Akaike Information Criteria; $\sigma_{\mathbf{u}}^{2}$, $\sigma_{\mathbf{m}}^{2}$, $\sigma_{\mathbf{q}}^{2}$, $\sigma_{\boldsymbol{\xi}}^{2}$, $\sigma_{\mathbf{e}}^{2}$, $h_{\mathbf{u}}^{2}$, $h_{\mathbf{m}}^{2}$, $q^{2}$, and $h_{\boldsymbol{\xi}}^{2}$ are the additive genetic, maternal genetic, common environment, transgenerational epigenetic, residual, and phenotypic variances, and additive genetic heritability, maternal genetic heritability, fraction of the phenotypic variance explained by the common environment effect, and transgenerational epigenetic heritability, respectively. ^b^Epi_genomic was used to emphasize that it is an epigenetic model including genomic information (no epigenomic information, e.g., DNA methylation) was used. Standard errors are presented within parenthesis.

**Table S4.** Variance components and genetic parameters for the back fat thickness of Landrace pigs using genetic and epigenetic models with pedigree- or single-step genomic BLUP based on the AIREML method.

| Parameter^a^ | Genetic | Epigenetic ($\lambda=0.20$) | Genomic | Epi_geno ($\lambda=0.40$)^b^ |
| --- | --- | --- | --- | --- |
| N iterations | 42 | 15 | 13 | 16 |
| AIC | -47565.917 | -47583.193 | -65667.237 | -65538.279 |
| $\sigma_{\mathbf{u}}^{2}$ | 0.008 (0.001) | 0.006 (0.001) | 0.008 (0.001) | 0.003 (0.001) |
| $\sigma_{\mathbf{m}}^{2}$ | 0.001 (0.000) | 0.001 (0.000) | 0.001 (0.000) | 0.000 (0.001) |
| $\sigma_{\mathbf{u,m}}$ | -0.001 (0.000) | -0.001 (0.000) | -0.001 (0.000) | 0.001 (0.001) |
| $\sigma_{\boldsymbol{\xi}}^{2}$ | - | 0.006 (0.003) | - | 0.007 (0.001) |
| $\sigma_{\mathbf{e}}^{2}$ | 0.008 (0.000) | 0.003 (0.002) | 0.008 (0.000) | 0.005 (0.001) |
| $\sigma_{P}^{2}$ | 0.015 (0.000) | 0.015 (0.000) | 0.015 (0.000) | 0.017 (0.001) |
| $h_{\mathbf{u}}^{2}$ | 0.491 (0.041) | 0.410 (0.054) | 0.498 (0.037) | 0.172 (0.061) |
| $h_{\mathbf{m}}^{2}$ | 0.045 (0.018) | 0.037 (0.017) | 0.044 (0.013) | 0.029 (0.043) |
| $r_{\mathbf{u}\boldsymbol{,}\mathbf{m}}$ | -0.476 (0.145) | -0.463 (0.281) | -0.537 (0.092) | 0.459 (1.573) |
| $h_{\boldsymbol{\xi}}^{2}$ | - | 0.393 (0.172) | - | 0.447 (0.061) |

^a^N iterations = number of iterations needed before model convergence; AIC = Akaike Information Criteria; $\sigma_{\mathbf{u}}^{2}$, $\sigma_{\mathbf{m}}^{2}$, $\sigma_{\mathbf{u,m}}$, $\sigma_{\boldsymbol{\xi}}^{2}$, $\sigma_{\mathbf{e}}^{2}$, $h_{\mathbf{u}}^{2}$, $h_{\mathbf{m}}^{2}$, $r_{\mathbf{u}\boldsymbol{,}\mathbf{m}}$, and $h_{\boldsymbol{\xi}}^{2}$ are the additive genetic, maternal genetic, covariance between additive and maternal genetic, common environment, transgenerational epigenetic, residual, and phenotypic variances, and additive genetic heritability, maternal genetic heritability, correlation between additive and maternal genetic effects, fraction of the phenotypic variance explained by the common environment effect, and transgenerational epigenetic heritability, respectively. ^b^Epi_genomic was used to emphasize that it is an epigenetic model including genomic information (no epigenomic information, e.g., DNA methylation) was used. Standard errors are presented within parenthesis.

**Table S5.** Variance components and genetic parameters for the total number born of Landrace pigs using genetic and epigenetic models with pedigree- or single-step genomic BLUP based on the AIREML method.

| Parameter^a^ | Genetic | Epigenetic ($\lambda=0.05$) | Genomic | Epi_geno ($\lambda=0.05$)^b^ |
| --- | --- | --- | --- | --- |
| N iterations | 15 | 9265 | 16 | 5466 |
| AIC | 52,845.104 | 52,847.104 | 52,795.728 | 52,831.772 |
| $\sigma_{\mathbf{u}}^{2}$ | 0.539 (0.133) | 0.539 (0.131) | 0.711 (0.119) | 2.634 (0.488) |
| $\sigma_{\mathbf{pe}}^{2}$ | 1.071 (0.177) | 1.069 (0.175) | 0.956 (0.158) | 0.827 (0.180) |
| $\sigma_{\boldsymbol{\xi}}^{2}$ | - | 0.002 (0.003) | - | 0.001 (0.002) |
| $\sigma_{\mathbf{e}}^{2}$ | 9.809 (0.185) | 9.809 (0.184) | 9.787 (0.184) | 9.778 (0.184) |
| $\sigma_{P}^{2}$ | 11.418 (0.167) | 11.418 (0.165) | 11.454 (0.171) | 13.240 (0.409) |
| $h_{\mathbf{u}}^{2}$ | 0.047 (0.011) | 0.047 (0.011) | 0.062 (0.010) | 0.199 (0.031) |
| $\mathrm{pe}^{2}$ | 0.141 (0.013) | 0.141 (0.013) | 0.146 (0.013) | 0.261 (0.024) |
| $h_{\boldsymbol{\xi}}^{2}$ | - | 0.000 (0.000) | - | 0.000 (0.000) |

^a^N iterations = number of iterations to converge; AIC = Akaike Information Criteria; $\sigma_{\mathbf{u}}^{2}$,
$\sigma_{\mathbf{pe}}^{2}$, $\sigma_{\boldsymbol{\xi}}^{2}$, $\sigma_{\mathbf{e}}^{2}$, $h_{\mathbf{u}}^{2}$, $\mathrm{pe}^{2}$, and $h_{\boldsymbol{\xi}}^{2}$ are the additive genetic, permanent environment, transgenerational epigenetic, residual, and phenotypic variances, and additive genetic heritability, fraction of the phenotypic variance explained by the permanent environment effect, and transgenerational epigenetic heritability, respectively. ^b^Epi_genomic was used to emphasize that it is an epigenetic model including genomic information (no epigenomic information, e.g., DNA methylation) was used. Standard error are within parenthesis.

**Table S6.** Variance components and genetic parameters for the number of piglets born alive of Landrace pigs using genetic and epigenetic models with pedigree- or single-step genomic BLUP based on the AIREML method.

| Parameter^a^ | Genetic | Epigenetic ($\lambda=0.10$) | Genomic | Epi_geno ($\lambda=0.10$)^b^ |
| --- | --- | --- | --- | --- |
| N iterations | 16 | 8671 | 13 | 17 |
| AIC | 51,802.931 | 51,804.931 | 51,748.476 | 51,791.723 |
| $\sigma_{\mathbf{u}}^{2}$ | 0.546 (0.128) | 0.544 (0.106) | 0.661 (0.108) | 2.428 (0.438) |
| $\sigma_{\mathbf{pe}}^{2}$ | 0.876 (0.162) | 0.855 (0.139) | 0.797 (0.141) | 0.687 (0.160) |
| $\sigma_{\boldsymbol{\xi}}^{2}$ | - | 0.022 (0.008) | - | 0.003 (0.003) |
| $\sigma_{\mathbf{e}}^{2}$ | 8.871 (0.167) | 8.871 (0.163) | 8.855 (0.166) | 8.847 (0.166) |
| $\sigma_{P}^{2}$ | 10.293 (0.151) | 10.293 (0.147) | 10.314 (0.154) | 11.965 (0.368 |
| $h_{\mathbf{u}}^{2}$ | 0.053 (0.012) | 0.053 (0.010) | 0.064 (0.010) | 0.203 (0.031) |
| $\mathrm{pe}^{2}$ | 0.138 (0.013) | 0.136 (0.012) | 0.141 (0.013) | 0.260 (0.024) |
| $h_{\boldsymbol{\xi}}^{2}$ | - | 0.002 (0.001) | - | 0.000 (0.000) |

^a^N iterations = number of iterations to converge; AIC = Akaike Information Criteria; $\sigma_{\mathbf{u}}^{2}$,
$\sigma_{\mathbf{pe}}^{2}$, $\sigma_{\boldsymbol{\xi}}^{2}$, $\sigma_{\mathbf{e}}^{2}$, $h_{\mathbf{u}}^{2}$, $\mathrm{pe}^{2}$, and $h_{\boldsymbol{\xi}}^{2}$ are the additive genetic, permanent environment, transgenerational epigenetic, residual, and phenotypic variances, and additive genetic heritability, fraction of the phenotypic variance explained by the permanent environment effect, and transgenerational epigenetic heritability, respectively. ^b^Epi_genomic was used to emphasize that it is an epigenetic model including genomic information (no epigenomic information, e.g., DNA methylation) was used. Standard errors are presented within parenthesis.
